# Exploring the Common Genetic Underpinnings of Chronic Pulmonary Disease and Esophageal Carcinoma Susceptibility

**DOI:** 10.7150/jca.95437

**Published:** 2024-04-29

**Authors:** Dengfeng Zhang, Yu zhou, Tianxing Lu, Jing Li, Longyu Zhu, Shujun Li, Yishuai Li, Xiaoliang Duan

**Affiliations:** 1 Department of Thoracic Surgery, The Second Hospital of Hebei Medical University, Shijiazhuang, China.; 2 Department of Thoracic Surgery, Hebei Chest Hospital, Shijiazhuang, China.; 3 Department of Radiotherapy, The Fourth Hospital of Hebei Medical University, Shijiazhuang, China.; 4 Hebei Provincial Key Laboratory of Pulmonary Diseases, Shijiazhuang, China.

**Keywords:** Esophageal cancer, Pulmonary disease, Genome-wide association study, Genetic correlation, Mendelian randomization analysis

## Abstract

**Background:** Pulmonary diseases and esophageal cancer are highly prevalent conditions with rising incidence worldwide. Prior evidence supports shared environmental and behavioral factors, but less is known regarding potential genetic links underlying this comorbidity. This study aimed to elucidate the complex genetic relationship between chronic lung diseases and esophageal cancer risk.

**Methods:** Linkage disequilibrium score regression assessed the genetic correlation between esophageal cancer and asthma, COPD, and idiopathic pulmonary fibrosis leveraging extensive GWAS datasets. Pleiotropic analysis, gene-set enrichment, eQTL mapping, and mendelian randomization causality analyses were then conducted to identify specific shared genetic variants, enriched pathways, causal relationships and gene regulatory mechanisms connecting lung disease and cancer susceptibility.

**Results:** Significant genetic correlations were observed between esophageal cancer and both COPD and asthma, but not idiopathic pulmonary fibrosis. Further analyses identified 13 pleiotropic loci and 6 shared genes including CHRNA4, ERBB3, and SMAD3, as well as pathways related to immune function. eQTL integration highlighted 53 genes like SOCS1, FGF2, and CHRNA5 with tissue-specific regulatory effects on disease risk. Bidirectional relationships were noted, whereby genetic predisposition to asthma and COPD increased esophageal cancer risk, while cancer liability reciprocally raised pulmonary fibrosis risk.

**Conclusions:** These genomic analyses provide initial evidence that shared genetic factors may underpin the comorbidity between lung conditions and esophageal malignancy. The genes and pathways identified offer insights into biological mechanisms linking both diseases, aiding future screening, prevention and therapeutic efforts to mitigate this growing comorbidity burden.

## Introduction

Esophageal cancer and chronic pulmonary diseases such as chronic obstructive pulmonary disease (COPD), idiopathic pulmonary fibrosis (IPF) and asthma are highly prevalent conditions associated with substantial morbidity and mortality burdens worldwide 1-4. Prior observational studies have noted epidemiologic links and clinical comorbidity between these diseases, suggesting potential common environmental or behavioral factors 5-7. For instance, smoking and inflammation influence pathogenesis of both malignancy and lung disorders 7-11. However, despite recognized shared risk determinants, the contributions of genetic factors underlying this medical comorbidity have remained less explored.

Recent advances in genomics research have enabled large-scale analyses assessing genetic contributions through methodologies including genome-wide association studies (GWAS), linkage disequilibrium score regression, Mendelian randomization, and various bioinformatic annotation approaches 12-16. By integrating multiple genetic correlation and causality assessment techniques with functional genomic annotation, these cutting-edge analytic frameworks now allow comprehensive interrogation of potential biological mechanisms driving comorbidity from sequence variation through to gene regulatory effects 17-19.

Leveraging extensive GWAS datasets, this study therefore aimed to elucidate the complex genetic relationship between chronic pulmonary diseases and esophageal carcinoma risk. We specifically sought to determine genetic correlation, identify pleiotropic loci, assess causality, and define regulatory mechanisms that may reciprocally associate lung conditions with esophageal cancer susceptibility. Delineating these intricate genomic links facilitating comorbidity may aid future screening, prevention, and therapeutic efforts to mitigate this growing global health burden.

## Methods

### Lung disease data

Pulmonary disease data were derived from a collaborative network of 23 biobank repositories across 4 continents as part of the Global Biobank Meta-analysis Initiative, comprising over 2.2 million individuals. Specifically, data on idiopathic pulmonary fibrosis were obtained from a large-scale meta-analysis 20 including 11,160 cases and 1,364,410 controls, with inverse-variance weighted fixed effects meta-analyses conducted for all ancestries, each ancestry, and sex-stratified ancestries across all biobanks. Similarly, the asthma GWAS was conducted as part of the Global Biobank Meta-analysis Initiative, performing a large-scale GWAS via meta-analysis of 22 biobanks across multiple ancestries, identifying 179 asthma-associated loci, 49 of which were novel. COPD data were directly accessed from the Global Biobank Meta-analysis Initiative, comprising 58,559 European ancestry cases and 937,358 controls. Additionally, for the asthma and IPF data, we only selected individuals of European ancestry for subsequent genetic analyses **(Table S1)**.

### Esophageal cancer data

Esophageal cancer data were derived from a meta-analysis of genome-wide association studies (GWAS) across four independent studies in Europeans, North Americans, and Australians 21. All cases were of European ancestry with histopathologically confirmed disease. The meta-analysis was conducted using fixed-effects inverse variance-weighted methods, comprising 4,112 esophageal adenocarcinoma cases and 17,159 representative controls from European, North American, and Australian GWAS **(Table S1)**. Stringent SNP quality control was implemented: (i) non-biallelic SNPs and those with ambiguous alleles were excluded; (ii) SNPs without rs-identifiers were removed; (iii) duplicate SNPs or those not present in 1000 Genomes or with allele mismatches were deleted; (iv) due to complex LD structure, SNPs within the major histocompatibility complex region (chr6: 28.5-33.5Mb) were excluded for LDSC analyses; (v) SNPs with minor allele frequency (MAF) < 0.01 were retained.

### Genetic correlation analysis

Linkage disequilibrium score regression (LDSC) 22 and high-density linkage disequilibrium (HDL) 23 methods were utilized to assess shared polygenic architecture between traits, with LD scores calculated from European ancestry samples in the 1,000 Genomes Project phase 3 as the reference panel 24; the HDL reference set comprised 1,029,876 quality-controlled HapMap3 SNPs.

We further utilized stratified LD score regression with data from different immune cell types to examine SNP heritability enrichment for esophageal cancer and pulmonary diseases, assessing whether specific cell types in these tissues demonstrated significant genetic enrichment. Data for 292 immune cell types (including B cells, gamma delta T cells, alpha beta T cells, innate lymphocytes, myeloid cells, stromal cells, and stem cells) were obtained from the ImmGen Consortium 25. After adjusting for the baseline model and all gene sets, we used the p-value of the z-score of the regression coefficients to evaluate significance of the SNP heritability enrichment estimates for each tissue and cell type.

### Pleiotropic analysis under composite null hypothesis (PLACO)

PLACO is a novel SNP-level approach that can investigate pleiotropic loci between complex traits using only summary-level genotype-phenotype association statistics 26. We computed the squared Z-scores for each variant and removed SNPs with extremely high Z2 (>80). Additionally, considering potential correlations between pulmonary diseases and esophageal cancer, we estimated the correlation matrix of Z. The omnibus test of pleiotropy was then performed using the level-α horizontal intersection-union test (IUT) method. The final p-value for the IUT test was the maximum of the p-values for testing H0 versus H1.

Based on the PLACO results, we further mapped the identified loci to nearby genes to explore potential shared biological mechanisms for these pleiotropic variants. We performed MAGMA Generalized Gene-Set Analysis of GWAS Data on genes overlapping or in proximity to pleiotropic loci identified by PLACO 27, to identify candidate pathways enriched for pleiotropic effects, as well as tissue enrichment of pleiotropic genes. Functional mapping and annotation by FUMA genome-wide association study (GWAS) 28 were utilized to determine biological roles of pleiotropic loci. A series of pathway enrichment analyses mapping annotated genes were conducted based on the Molecular Signatures Database (MSigDB) to elucidate functionality 29. eQTL analyses incorporated SNP-gene association data including esophageal tissue, lung tissue, and whole blood.

### Mendelian randomization analysis

We used the clumping program in PLINK software (6) to select all loci independently associated with the disease at genome-wide significance (P<5×10^-8) as instrumental variables (IVs), with an r^2 threshold of 0.001 within a 10,000kb window. To ensure the strength of the IVs, we calculated the r^2 and F-statistic for each IV 30. The F-statistic was computed as: where r^2 represents the proportion of variance explained by the IV, n is the sample size, and k is the number of SNPs. The primary method adopted for Mendelian randomization (MR) was inverse variance weighted (IVW), which requires the IVs to satisfy 3 assumptions: (1) the IV should be associated with exposure; (2) the IV should not be associated with confounders of the exposure-outcome association; (3) the IV affects the outcome only through the exposure. We conducted several sensitivity analyses. First, the IVW and MR-Egger's Q test can detect potential violations of assumptions through heterogeneity between the IV estimates 31. Second, we applied MR-Egger to estimate directional pleiotropy according to its intercept, ensuring genetic variants are independently associated with exposure and outcome 32. We increased the stability and robustness of results through additional analyses using MR methods with different modeling assumptions and strengths (DIVW, MR-RAPS, weighted median and weighted mode). All statistical analyses were performed in R 3.5.3, with MR analyses utilizing the MendelianRandomization package 33.

## Results

**Figure 1:**
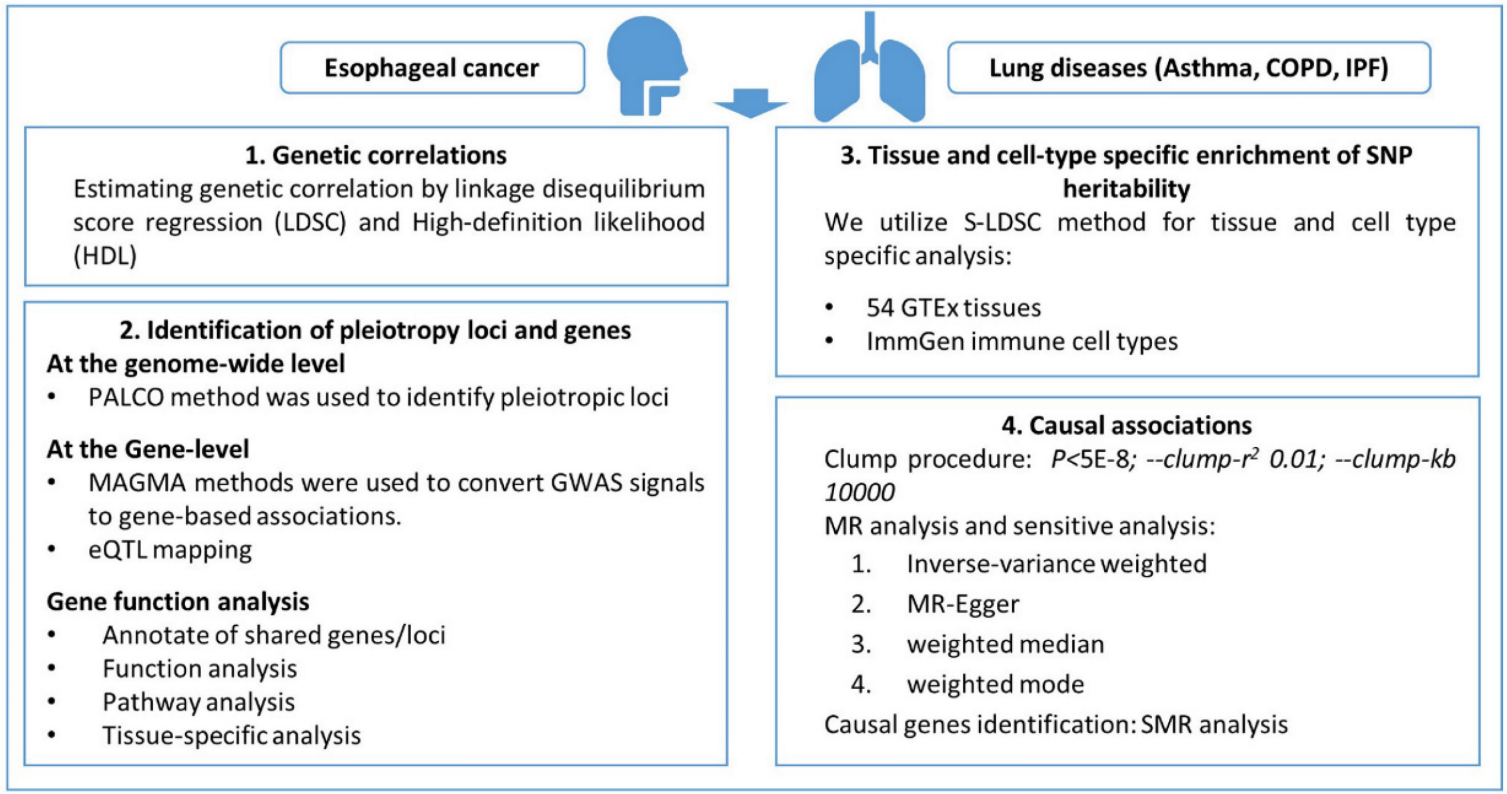
Flow diagram outlining the full analysis process.

A flow diagram outlining the full analysis process is provided in **Figure 1**.

### Genetic correlations

LDSC genetic correlation analyses revealed significant genetic correlations between pulmonary diseases (COPD and asthma) and esophageal cancer: COPD (rg = 0.217, P = 4.22E-5), asthma (rg = 0.158, P = 2.00E-4); however, the genetic correlation between idiopathic pulmonary fibrosis and esophageal cancer was not significant (rg = 0.071, P = 0.5761). HDL analysis also corroborated relationships between esophageal cancer and COPD (rg = 0.197, P = 4.27E-04) and asthma (rg = 0.143, P = 0.005) **(Table S2)**. The LDSC intercepts excluded the possibility of sample overlap.

### Identification of pleiotropy loci and genes

**Figure 2:**
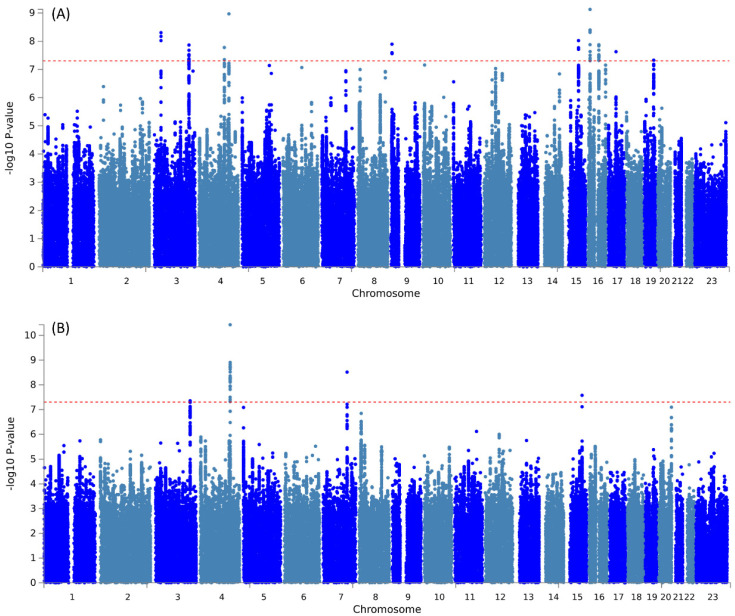
Manhattan plot illustrating the pleiotropic loci between pulmonary diseases and esophageal cancer.

**Figure 3:**
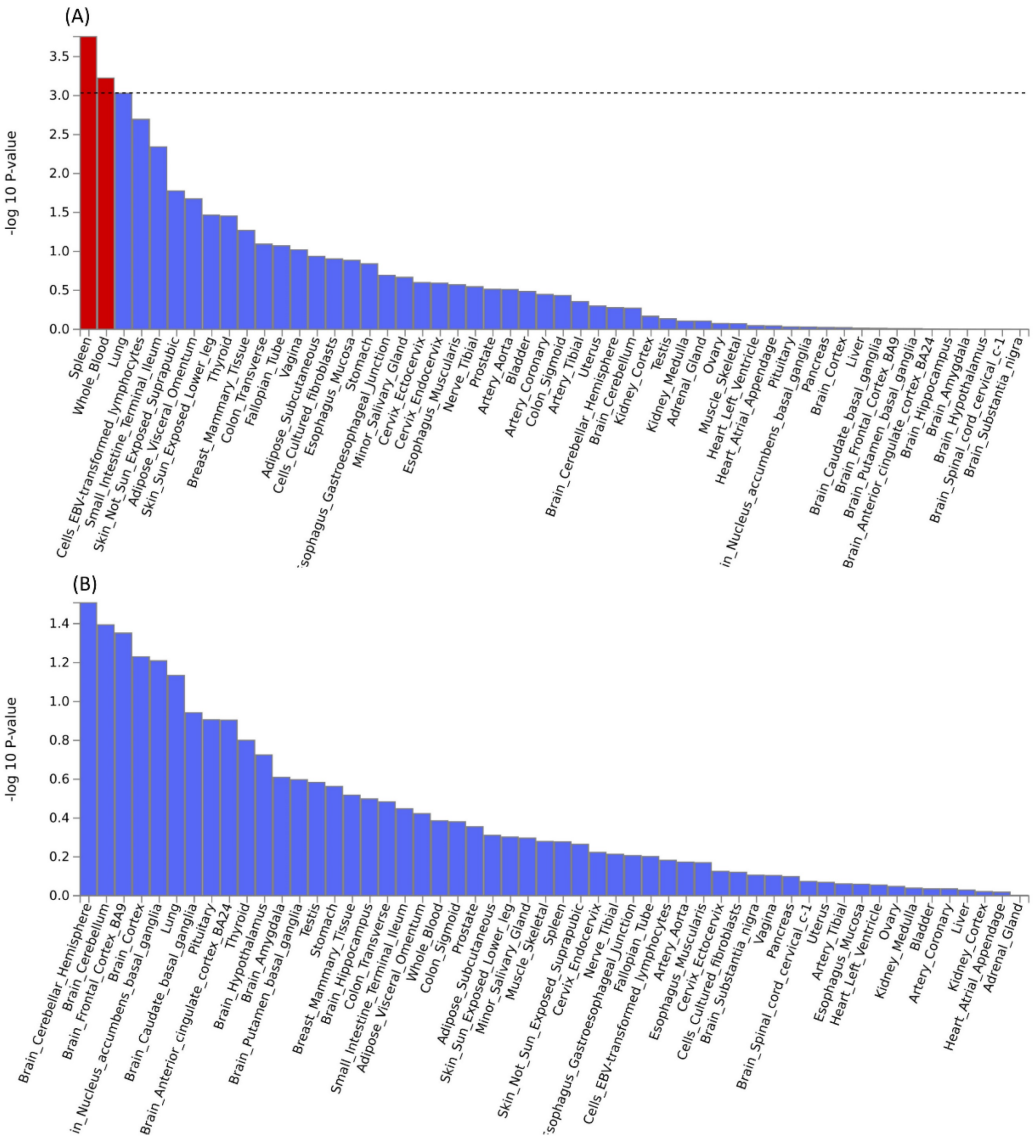
Tissue-specific analysis based on genome-wide pleiotropy using MAGMA.

Further pleiotropic analysis (PLACO) was undertaken between the two diseases, and the Manhattan plots are shown in** Figure 2**. As summarized in **Table 1**, we identified 13 pleiotropic loci (9 for asthma, 4 for COPD), and 4q31.21 was shared between the two phenotypes **(Table S3)**. Notably, the QQ plots showed no genomic inflation **(Figure S1)**. In addition, basic information for each genomic risk locus can be found in **Figure S2**. We identified that among the 9 genomic loci associated with asthma, the locus at 15:67395918-68218648 harbors the highest number of SNPs. Similarly, within the 4 genomic loci associated with COPD, the locus at 15:78712119-79080798 has the highest number of SNPs. Regarding the functional impacts of pleiotropic SNPs on genes, please refer to **Figure S3**. We observed that the functional impact of pleiotropic SNPs on genes is predominantly concentrated in intronic and intergenic regions. Specifically, the pleiotropic SNPs between asthma and esophageal cancer primarily affect gene function in intronic regions, whereas those between COPD and esophageal cancer exert their main influence on gene function in intergenic regions. Finally, regional plots for each risk locus are presented in **Figures S4 to S16**. Gene-set enrichment analysis using MAGMA on the pleiotropic results revealed the top 10 significantly enriched gene sets as shown in **Table 2**, involving pathways such as alpha beta T cell differentiation, alpha beta T cell activation, positive regulation of RNA metabolic process, chromatin, Foxp3 in COVID19 and positive regulation of transcription by RNA polymerase II. Moreover, tissue-specific MAGMA analysis indicated that the most significant enrichment evidence in both diseases appeared in spleen and whole blood tissues, which are closely related to immune processes **(Figure 3)**. Importantly, this section of MAGMA gene-set and tissue-specific analyses was based on the complete SNP p-value distribution.

By leveraging the location information of lead SNPs, we successfully mapped nearby genes associated with these pleiotropic risk loci **(Table 1)**. Subsequently, we conducted further MAGMA gene test and identified 6 significantly pleiotropic genes **(Figure S17 and Table S4)**: RIN3, SMAD3, AAGAB, ERBB3, LPP, and CHRNA4. Details on the nearby and MAGMA genes are provided in **Table S5**. The QQ plot **(Figure S18)** did not show genomic inflation, indicating the results are credible. The expression patterns of pleiotropic genes across different tissues are shown in **Figure S19**, demonstrating differential expression of these genes in some tissues including EBV transformed lymphocytes cells, whole blood, spleen, esophagus, etc. Tissue-specific enrichments are detailed in **Figure S20**. Pathway analysis results can be found in **Figure S21**, while enrichments for cell types are shown in **Figure S22**. The key pathways involved include: the receptor complex, regulation of chemotaxis, the nuclear speckle, and regulation of growth.

**Figure 4:**
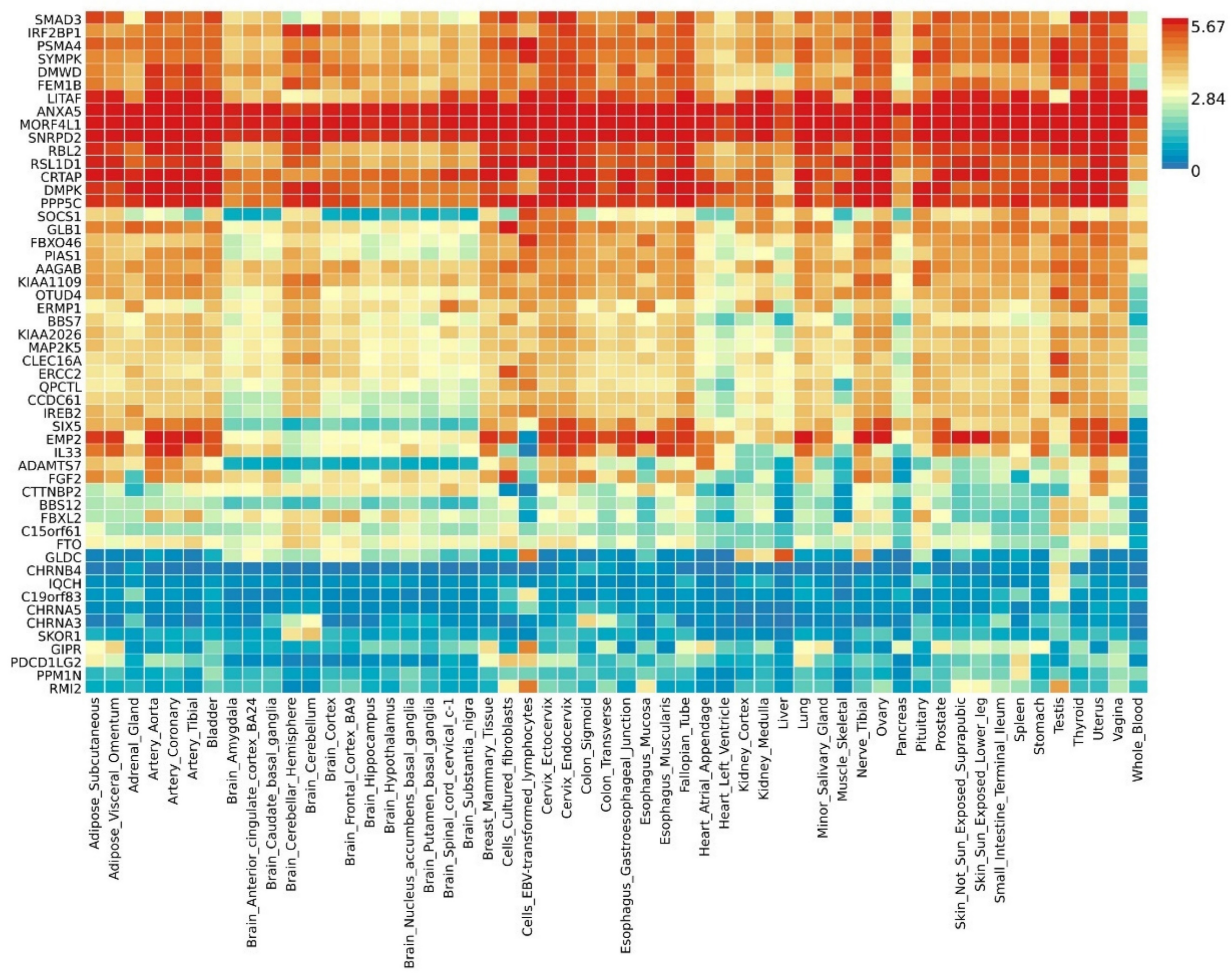
Expression profiles of pleiotropic eQTL genes across different tissues.

**Figure 5:**
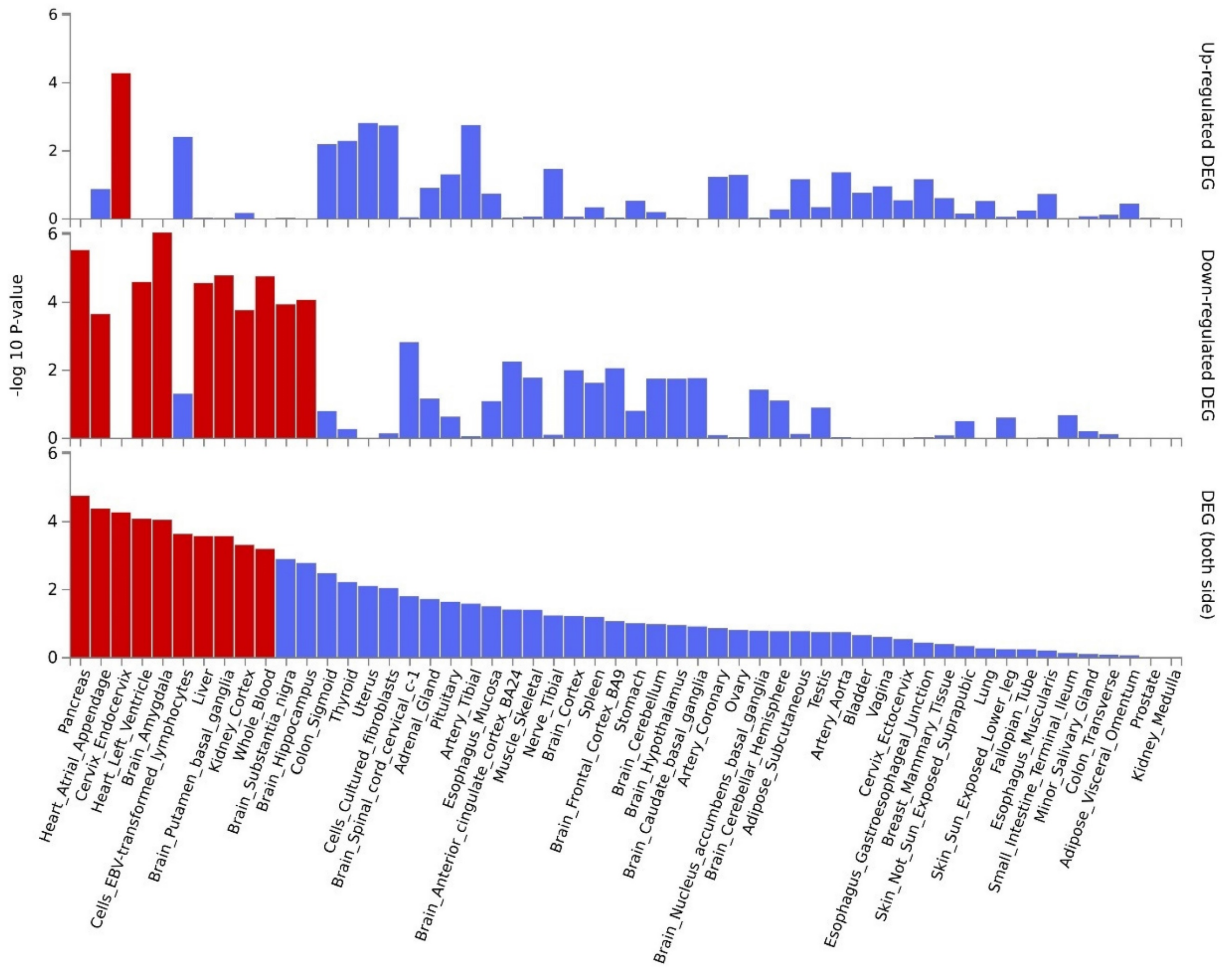
Tissue-specific enrichment analysis based on pleiotropic eQTL genes.

By further leveraging eQTL information (including esophageal tissue, lung tissue, spleen, and whole blood data), we successfully identified eQTL genes associated with these pleiotropic risk loci **(Table S6)**. A total of 53 relevant genes were identified, including several widely-studied molecules such as SMAD3, SOCS1, ERCC2, IL33, FGF2, FTO, CHRNA5, and PPM1N, with 44 genes related to asthma and 9 genes related to chronic obstructive pulmonary disease (COPD). Expression patterns of pleiotropic eQTL genes across tissues can be found in **Figure 4**. In various tissues, notable expression is observed for genes such as LITAF, ANXA5, MORF4L1, and SNRPD2. Conversely, genes including CHRNB4, IQCH, C19orf83, and CHRNA5 exhibit significantly reduced expression across multiple tissues. Enrichments of these genes in different tissues are detailed in **Figure 5**, showing significant enrichments in whole blood, esophagus colon, lung, brain, skeletal muscle, etc. Pathway enrichment analysis results can be found in **Figure S23** and **Figure S24**. Enrichment analysis implicated various pathways including behavioral response to nicotine, fat cell differentiation, the nuclear speckle, response to fatty acids, myeloid cell differentiation, and others.

### Tissue and cell-type specific enrichment of SNP heritability

We subsequently utilized stratified linkage disequilibrium score regression (S-LDSC) to analyze genetics data specific to particular tissue or cell types. This approach estimates the genetic contribution of different cell types or tissues, which aids in pinpointing the source of genetic associations with traits or diseases. It enables interpretation of genome-wide polygenic signals (GWAS) in the context of a particular tissue or cell type by elucidating in which tissue or cell type genetic variation has a functional effect, thereby furthering understanding of the biological mechanisms underlying complex traits and diseases.

**Figure 6:**
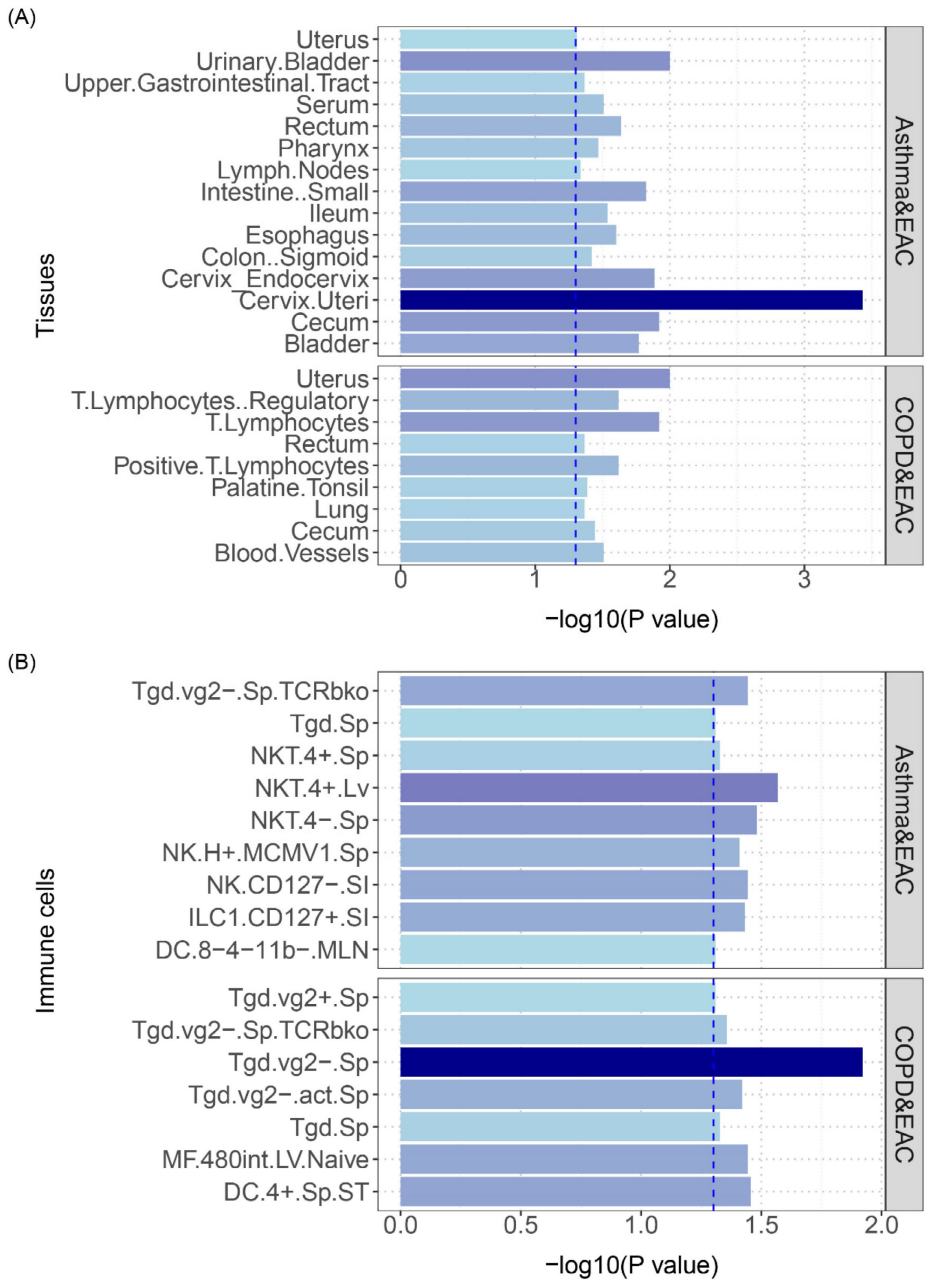
(A) S-LDSC enrichment analysis identifies tissues with significant heritability enrichment between pulmonary diseases and esophageal cancer. (B) S-LDSC enrichment analysis identifies immune cells with significant heritability enrichment between pulmonary diseases and esophageal cancer. The blue dashed line represents the significance threshold of 0.05.

The analysis results are shown in **Figure 6**: asthma and COPD had overlapping polygenicity for EAC in the uterus and lymphocyte tissues, with the cervical tissue having the highest significance; additionally, asthma and COPD showed overlapping polygenicity for EAC in NK and Tgd cells, with Tgd cells having the highest significance; asthma and EAC exhibited the most enrichment in NK cells, indicating that NK cells play a vital role in the developmental processes of both diseases; COPD and EAC displayed the most enrichment in Tgd cells, signifying that this cell type is critical in the disease courses of both (specific results are shown in **Table S7** and **Table S8).**

### Causal associations

Finally, we performed causal inference on the relationship between lung diseases and esophageal cancer using two-sample MR methods, which supported the significant association between the two, with genetic instruments used listed in **Table S9**. Sensitivity analyses are shown in **Table 3**, and full sensitivity analysis results are in **Table S10**.

Causal effect analysis of asthma on esophageal cancer showed consistent results across three MR methods (IVW, DIVW, MR-RAPS) that asthma has a causal effect on esophageal cancer. The heterogeneity test P was 0.189, indicating no heterogeneity existed. The MR-Egger intercept P>0.05, suggesting no influence from horizontal pleiotropy. Reverse causal effect analysis of esophageal cancer on asthma also showed significant causal effects between the two, with consistent results from three MR methods (IVW, DIVW, MR-RAPS). The heterogeneity test P was 0.297, indicating no heterogeneity. The bidirectional causal relationships further validate potential pleiotropy between the two diseases. Additionally, causal effect analysis of esophageal cancer on IPF showed consistent results from three MR methods (IVW, DIVW, MR-RAPS) that esophageal cancer has a causal effect on IPF. The heterogeneity test P was 0.831, indicating no heterogeneity existed.

## Discussion

In this large-scale analysis, we identified significant genetic correlations and pleiotropic loci between esophageal cancer and pulmonary diseases including COPD and asthma by leveraging GWAS summary statistics. Enrichment analysis implicated various pathways including behavioral response to nicotine, fat cell differentiation, the nuclear speckle, response to fatty acids, myeloid cell differentiation, and others, which may be involved in the pleiotropic mechanisms between diseases. Causal inferences supported bidirectional relationships, where genetic liability for asthma and COPD increased esophageal cancer risk, while liability for esophageal cancer conversely increased asthma and IPF susceptibility. Our integrated approach combining multiple state-of-the-art methodologies provides robust evidence for potential shared genetic components across these diseases.

Prior observational studies have noted epidemiologic connections between esophageal carcinoma and chronic respiratory illnesses including COPD, IPF, and asthma, suggesting potential shared environmental or behavioral factors 34-38. For example, several large cohort studies found a significantly heightened risk of esophageal cancer among COPD patients compared to controls without airflow obstruction 5, 39. Likewise, population-based analyses have revealed a nearly 3-fold higher prevalence of asthma among esophageal cancer cases versus matched controls. Proposed common contributors to pathogenesis of both malignancy and lung disease include tobacco use, which damages the esophageal mucosa while promoting airway inflammation and remodeling 40-43. Additionally, systemic inflammatory states may facilitate esophageal carcinogenesis while also exacerbating pulmonary disease severity 44, 45. These epidemiologic observations provide clinical evidence complementing the genetic correlations identified here, further supporting that shared etiologic factors likely underpin the co-occurrence of these conditions. Elucidating the intricate biological mechanisms linking esophageal carcinoma and chronic lung diseases may contribute to improved prevention, screening and management for patients at high risk of this comorbidity.

Regarding the pleiotropic loci we identified, some of these loci have previously been reported in association with esophageal cancer or pulmonary diseases. For instance, the pleiotropic locus in the 15q25 region has been found to be associated with lung cancer and COPD 46-49. The pleiotropic locus at 4q31.21 has also been reported to influence susceptibility to asthma 50. In addition, some of the newly discovered pleiotropic loci, such as those located at 3p21.31 and 10q22.3, have not been previously reported in association with these two types of diseases. This may represent potential common genetic factors between the newly discovered esophageal cancer and pulmonary diseases in our study. Overall, these results provide further support for the shared genetic architecture between esophageal cancer and pulmonary diseases, contributing to our understanding of the relationship between these two conditions. Subsequent functional studies are needed to validate the impact of these newly discovered pleiotropic loci on diseases and to elucidate their potential mechanisms in the onset of diseases.

Our research findings suggest the potential existence of shared genetic mechanisms between esophageal cancer and pulmonary diseases. Enrichment analysis indicates that these pleiotropic loci involve various biological pathways, such as behavioral responses to nicotine 51-54, adipocyte differentiation 55, 56, nuclear speckles 57, 58, responses to fatty acids 59, 60, and granulocyte differentiation 61, among others. These pathways are associated with processes like smoking, inflammation, and immune function, contributing to an explanation of the interconnections between diseases. Additionally, tissue and cell-type analyses reveal that the genetic correlations between esophageal cancer and pulmonary diseases predominantly originate from cervical tissue, lymphocytes, NK cells, and Tgd cells, among others. This implies a potential pivotal role of these tissues and immune cells in the onset of diseases.

Our study had several key strengths. The utilization of immense GWAS sample sizes across diseases enhanced statistical power to uncover subtle shared genetic effects that may have been difficult to detect in smaller-scaled efforts, overcoming barriers like residual confounding that can distort observational studies. Additionally, the random assortment of genetic variants avoids reverse causation, a key advantage of MR designs. However, some limitations should be considered when interpreting the results. It is important to note that the predominantly European ancestry of the study participants may limit the generalizability of the findings to other populations. The available GWAS data were predominately of European ancestry, restricting generalizability, with further diversity needed to determine if findings extend to other populations. The use of summary statistics also precluded stratified analyses to pinpoint specific at-risk subgroups. While offering statistical power, the high genomic stringency of methods like PLACO may miss small-effect pleiotropic loci that contribute meaningfully to comorbidity overall. Even for significant hits, deciphering the precise biological functionality through which variants influence both traits remains challenging. By demonstrating shared genetic underpinnings across multiple independent large-scale GWAS, our work provides initial evidence towards biological mechanisms that may reciprocally link esophageal cancer and chronic lung conditions. Future research should explore whether lifestyle or pharmaceutical interventions modifying genetic risk could mitigate progression across both diseases. With further confirmation in diverse cohorts and functional validation, clinically translating findings could enable earlier targeted screening and prevention efforts for those most vulnerable to this comorbidity.

## Supplementary Material

Supplementary figures and tables.

## Figures and Tables

**Table 1:** Information on the thirteen identified pleiotropic loci.

Trait pairs	GenomicLocus	Chromosome: start-end	rsID	P	Mapped Genes
Asthma&EAC	3p22.3	3:32923640-33185407	rs6764245	4.97E-09	CCR4, GLB1
Asthma&EAC	3q26.2	3:168585686-168976021	rs879394	1.36E-08	RP11-368I23.4, RP11-152C17.1
Asthma&EAC	4q27	4:122884909-123727295	rs72687029	1.67E-08	KIAA1109
Asthma&EAC	4q31.21	4:145227600-146177041	rs1032296	1.07E-09	RP11-361D14.2, KRT18P51
Asthma&EAC	9p24.1	9:5652270-6585872	rs62556407	2.76E-08	KIAA2026
Asthma&EAC	15q22.33	15:67395918-68218648	rs2289791	9.50E-09	SMAD3
Asthma&EAC	16p13.13	16:11004363-11467218	rs12923849	7.46E-10	CLEC16A
Asthma&EAC	16q12.2	16:53579222-53848561	rs1558902	1.33E-08	FTO
Asthma&EAC	19q13.32	19:46150182-46470434	rs7256524	4.70E-08	FBXO46
COPD&EAC	3q26.2	3:168587408-168976021	rs13078090	4.44E-08	RP11-152C17.1, MECOM
COPD&EAC	4q31.21	4:145227600-146177041	rs1032296	3.72E-11	RP11-361D14.2, KRT18P51
COPD&EAC	7q31.2	7:116910447-117449242	rs10278953	3.06E-09	ASZ1
COPD&EAC	15q25.1	15:78712119-79080798	rs564585	2.66E-08	CHRNA5

**Table 2:** Results of gene set analysis (top 10), with significant results (P_bon_ < 0.05 after multiple corrections) highlighted in red.

Trait pairs	Gene Sets	N genes	Beta	SE	*P*	*P*_bon_
COPD&EAC	REACTOME_HIGHLY_CALCIUM_PERMEABLE_NICOTINIC_ACETYLCHOLINE_ RECEPTORS	9	1.515	0.336	3.38E-06	0.058
COPD&EAC	GOBP_BEHAVIORAL_RESPONSE_TO_NICOTINE	8	1.337	0.316	1.20E-05	0.204
COPD&EAC	REACTOME_HIGHLY_CALCIUM_PERMEABLE_POSTSYNAPTIC_NICOTINIC_ ACETYLCHOLINE_RECEPTORS	11	1.273	0.304	1.45E-05	0.246
COPD&EAC	REACTOME_VESICLE_MEDIATED_TRANSPORT	657	0.132	0.032	1.74E-05	0.297
COPD&EAC	GOBP_RESPONSE_TO_NICOTINE	44	0.574	0.140	1.98E-05	0.337
COPD&EAC	REACTOME_MEMBRANE_TRAFFICKING	618	0.127	0.033	5.44E-05	0.925
COPD&EAC	BROWNE_HCMV_INFECTION_4HR_DN	241	0.182	0.053	2.69E-04	1.000
COPD&EAC	DAZARD_UV_RESPONSE_CLUSTER_G2	25	0.588	0.173	3.50E-04	1.000
COPD&EAC	SA_PTEN_PATHWAY	17	0.690	0.206	4.13E-04	1.000
COPD&EAC	MULLIGHAN_NPM1_SIGNATURE_3_DN	156	0.223	0.067	4.24E-04	1.000
Asthma&EAC	GOBP_ALPHA_BETA_T_CELL_DIFFERENTIATION	116	0.461	0.088	7.24E-08	1.23E-03
Asthma&EAC	GOBP_ALPHA_BETA_T_CELL_ACTIVATION	165	0.358	0.073	5.27E-07	0.009
Asthma&EAC	GOBP_POSITIVE_REGULATION_OF_RNA_METABOLIC_PROCESS	1756	0.105	0.022	9.20E-07	0.016
Asthma&EAC	GOCC_CHROMATIN	1249	0.123	0.026	1.19E-06	0.020
Asthma&EAC	WP_FOXP3_IN_COVID19	15	1.251	0.266	1.30E-06	0.022
Asthma&EAC	GOBP_POSITIVE_REGULATION_OF_TRANSCRIPTION_BY_RNA_POLYMERASE_II	1187	0.123	0.027	1.65E-06	0.028
Asthma&EAC	ZWANG_CLASS_3_TRANSIENTLY_INDUCED_BY_EGF	215	0.296	0.065	2.94E-06	0.050
Asthma&EAC	GOBP_POSITIVE_REGULATION_OF_MACROMOLECULE_BIOSYNTHETIC_PROCESS	1836	0.097	0.022	3.01E-06	0.051
Asthma&EAC	GOMF_PURINE_NUCLEOTIDE_BINDING	1914	0.091	0.021	5.04E-06	0.086
Asthma&EAC	GOMF_TRANSCRIPTION_REGULATOR_ACTIVITY	1643	0.103	0.023	5.07E-06	0.086

**Table 3:** Outline of results of the Mendelian Randomization (MR) analysis.

Exposures	Outcomes	Methods	Estimate	P	Heterogeneity test	
					Estimate	P
Asthma	EAC	IVW	1.194 (1.048, 1.361)	0.008	131.336	0.189
		DIVW	1.198 (1.041, 1.378)	0.012		
		MR-RAPS	1.201 (1.039, 1.388)	0.013		
		Weighted mode	1.242 (0.892, 1.727)	0.199		
		Weighted median	1.225 (0.996, 1.508)	0.055		
		MR-Egger (slope)	1.182 (0.824, 1.696)	0.360		
		MR-Egger (intercept)	0 (-0.016, 0.017)	0.953		
EAC	Asthma	IVW	1.065 (1.022, 1.11)	0.003	1.088	0.297
		DIVW	1.067 (1.02, 1.117)	0.005		
		MR-RAPS	1.066 (1.018, 1.116)	0.007		
EAC	IPF	IVW	1.203 (1.019, 1.42)	0.029	0.046	0.831
		DIVW	1.21 (1.013, 1.445)	0.036		
		MR-RAPS	1.203 (1.008, 1.435)	0.040		
