# Supplementary material for: Exploring the Common Genetic Underpinnings of Chronic Pulmonary Disease and Esophageal Carcinoma Susceptibility
Source: J Cancer. 2024 Apr 29;15(11):3406–17. doi: 10.7150/jca.95437 (PMC11134432; doi:10.7150/jca.95437)

**Figure S1.** illustrates the QQ plot for the pleiotropy analysis. (A)The QQ plot for asthma and esophageal cancer. (B) The QQ plot forCOPD and esophageal cancer.


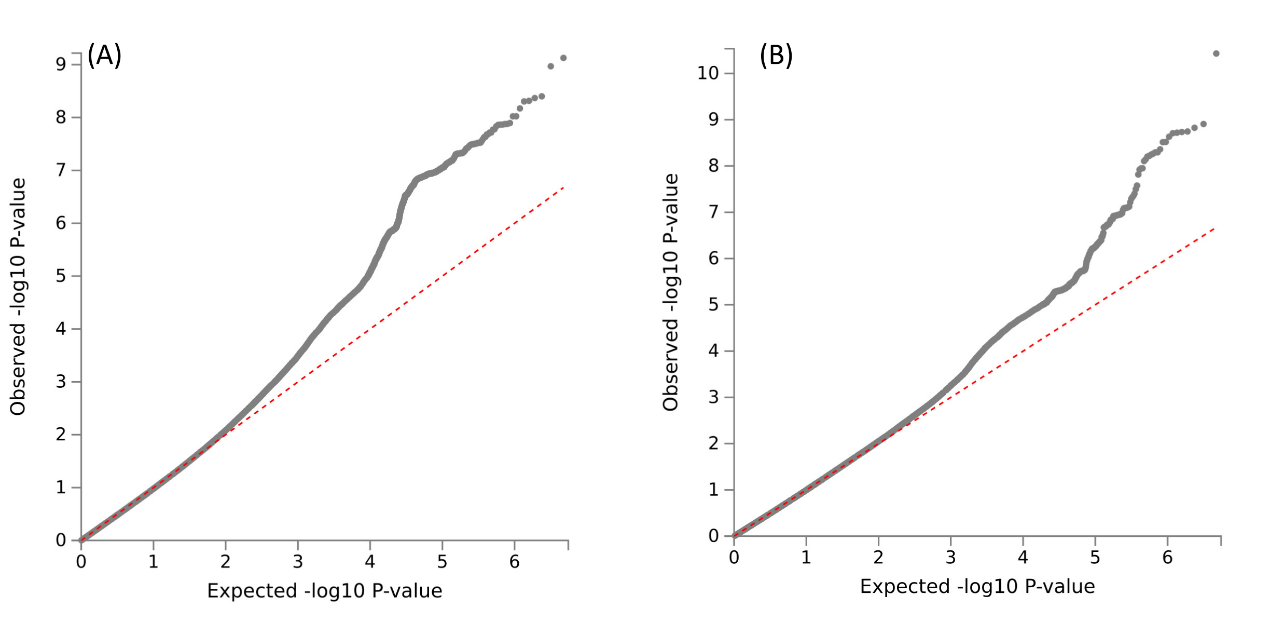


**Figure S2.** The summarized information for each genomic risk locus includes, from left to right, the size of the risk locus, the number of SNPs, the count of mapped genes, and the number of genes located within the locus. (A) Genomic risk locus between asthma and esophageal cancer. (B) Genomic risk locus between COPD and esophageal cancer.


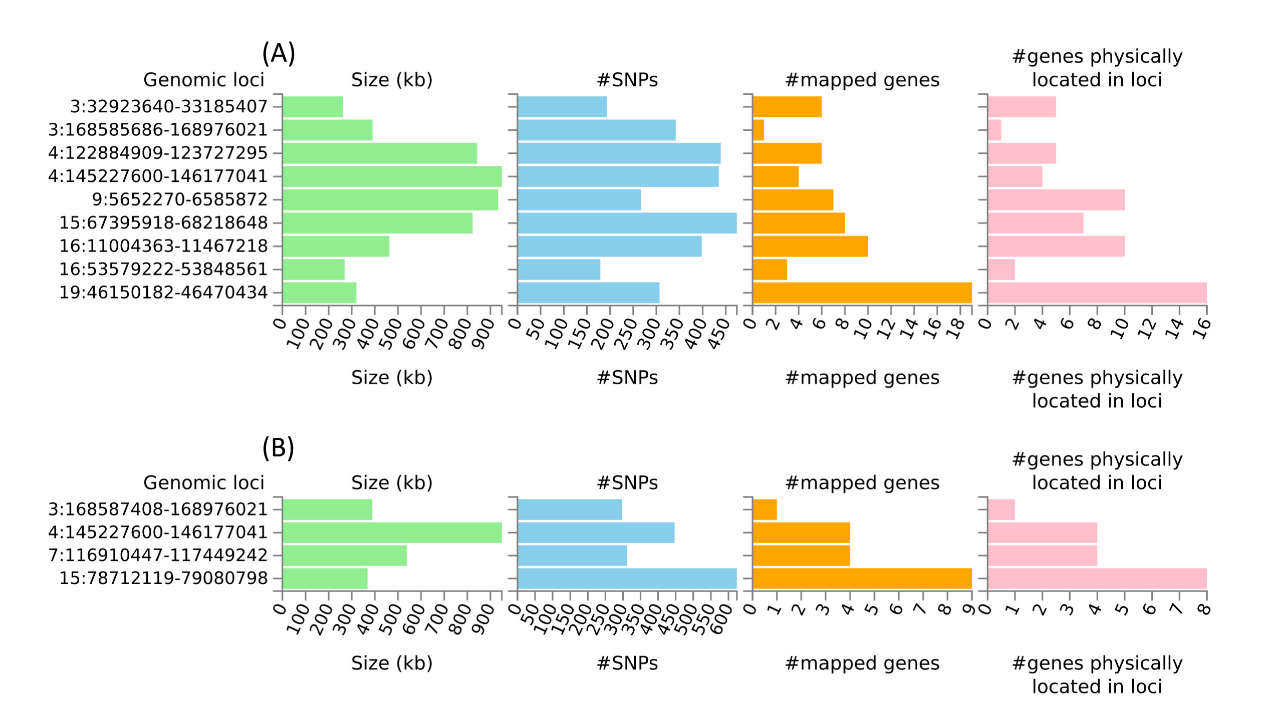


**Figure S3** The functional impact of pleiotropic SNPs on genes. (A) The pleiotropic SNPs between asthma and esophageal cancer. (B) The pleiotropic SNPs between COPD and esophageal cancer.


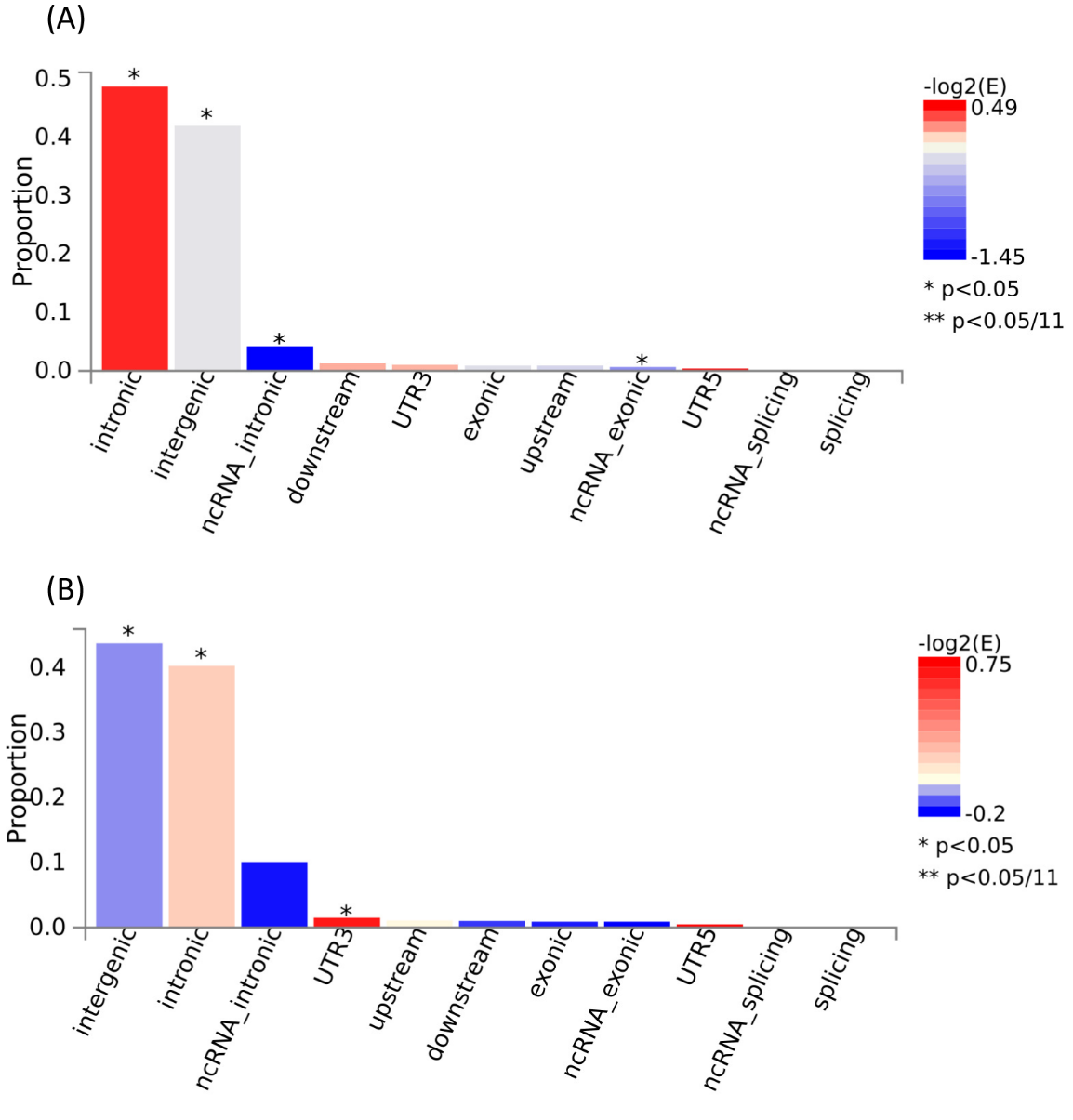


**Figure S4**. The regional plot for the shared locus of ASTHMA & EAC (rs6764245).


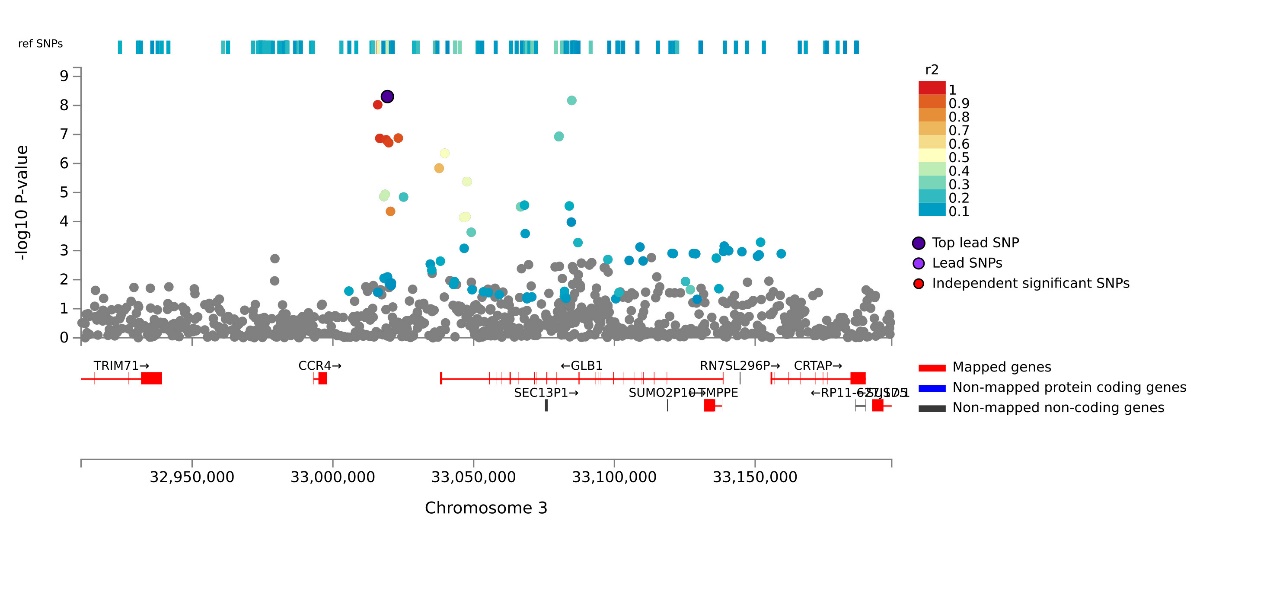


**Figure S5.** The regional plot depicting the shared genetic locus for ASTHMA and EAC, specifically focusing on rs879394.


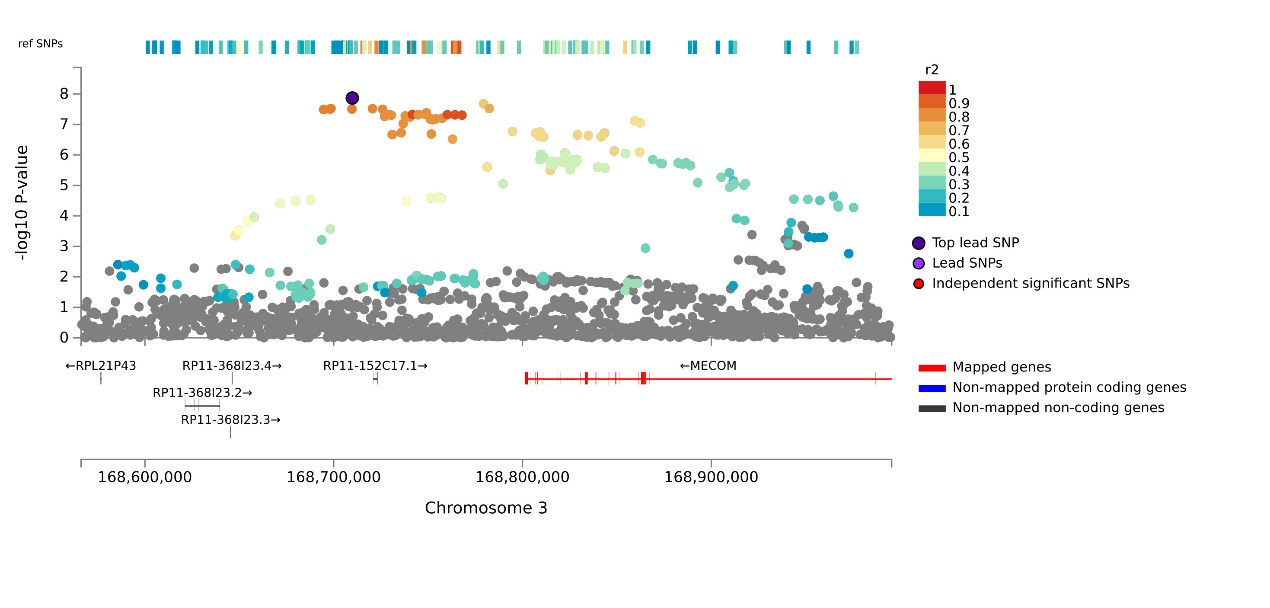


**Figure S6.** The regional plot illustrating the shared genetic locus for ASTHMA and EAC, with a specific emphasis on rs72687029.


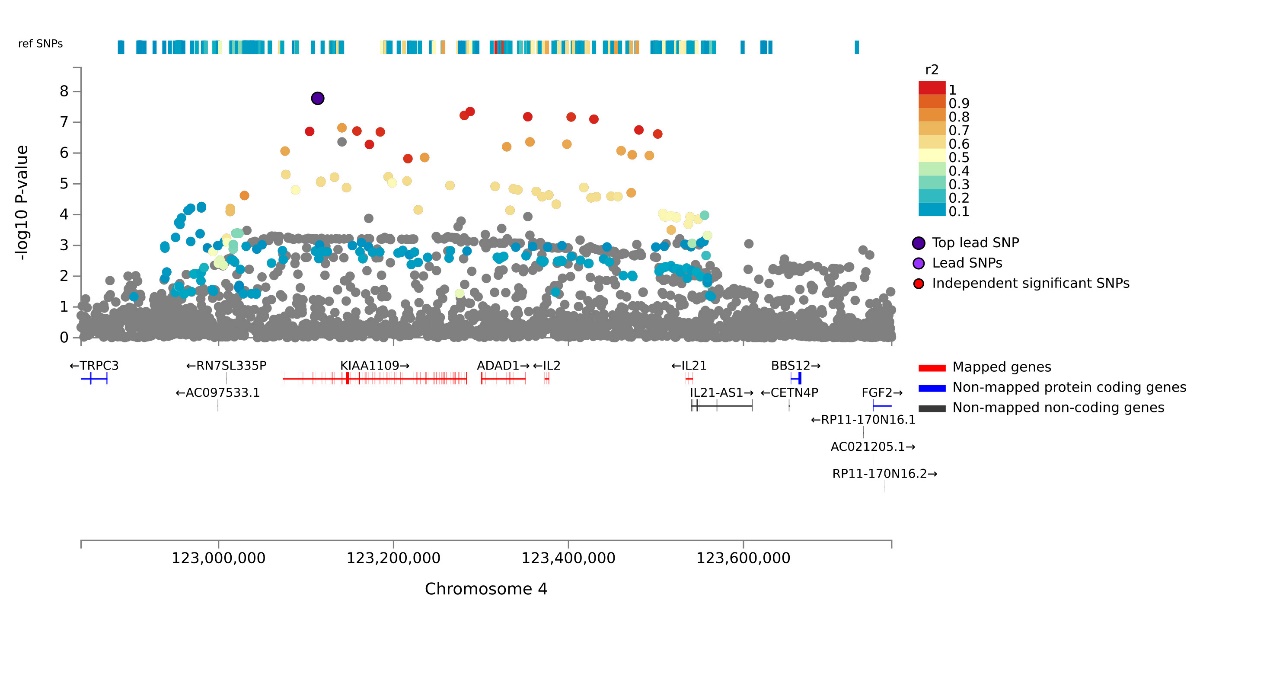


**Figure S7.** The regional plot highlighting the shared genetic locus for ASTHMA and EAC, with specific reference to rs1032296.


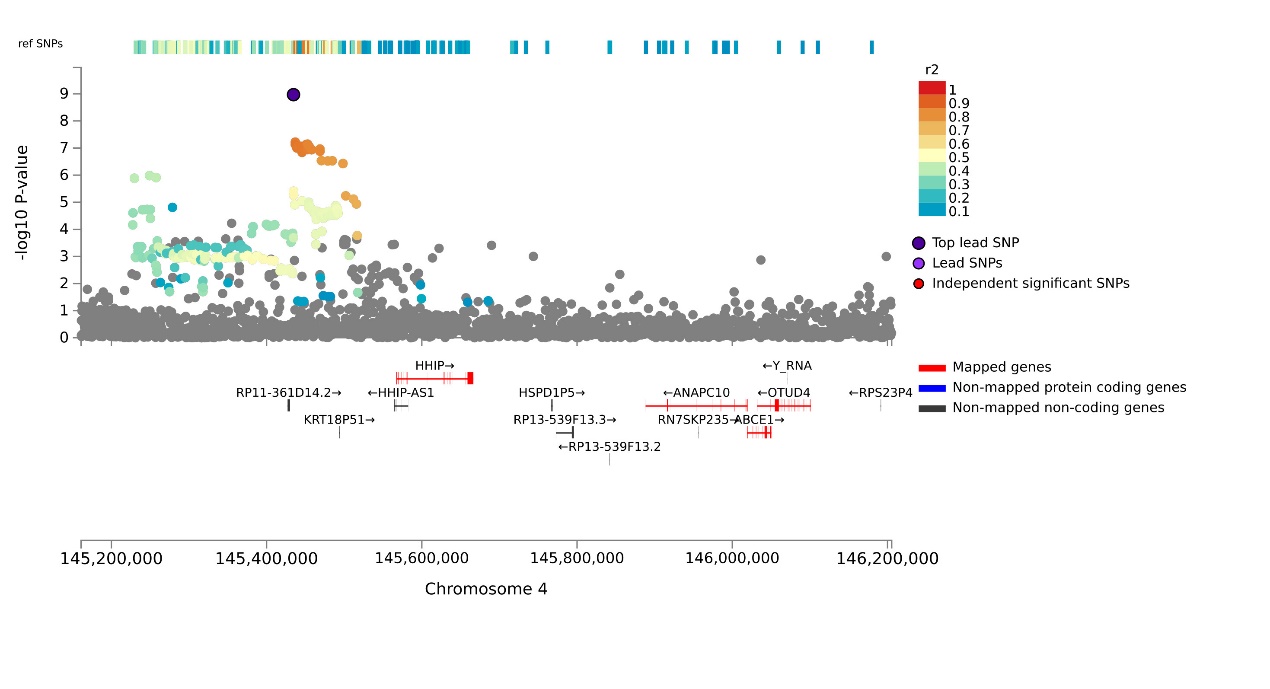


**Figure S8**. The regional plot delineating the shared genetic locus for ASTHMA and EAC, particularly focusing on rs62556407.


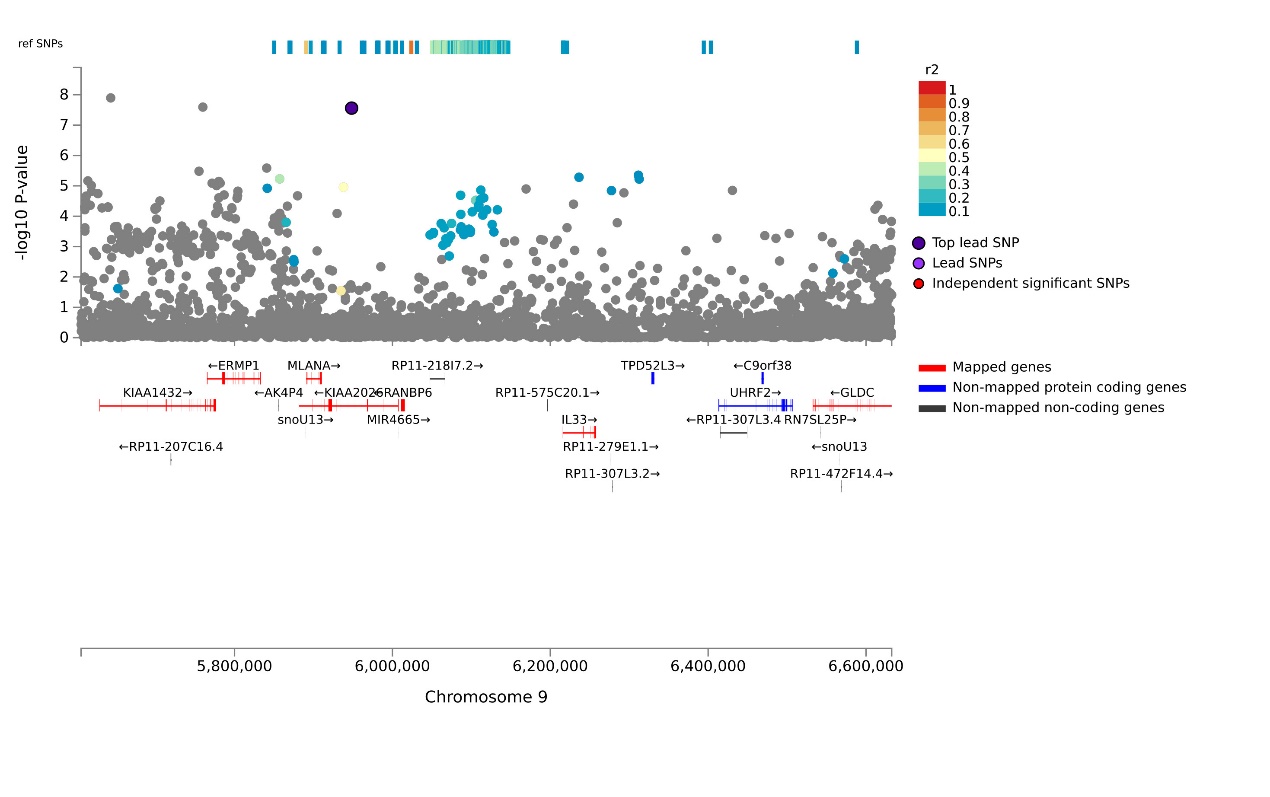


**Figure S9.** The regional plot illustrating the shared genetic locus for ASTHMA and EAC, specifically emphasizing rs2289791.


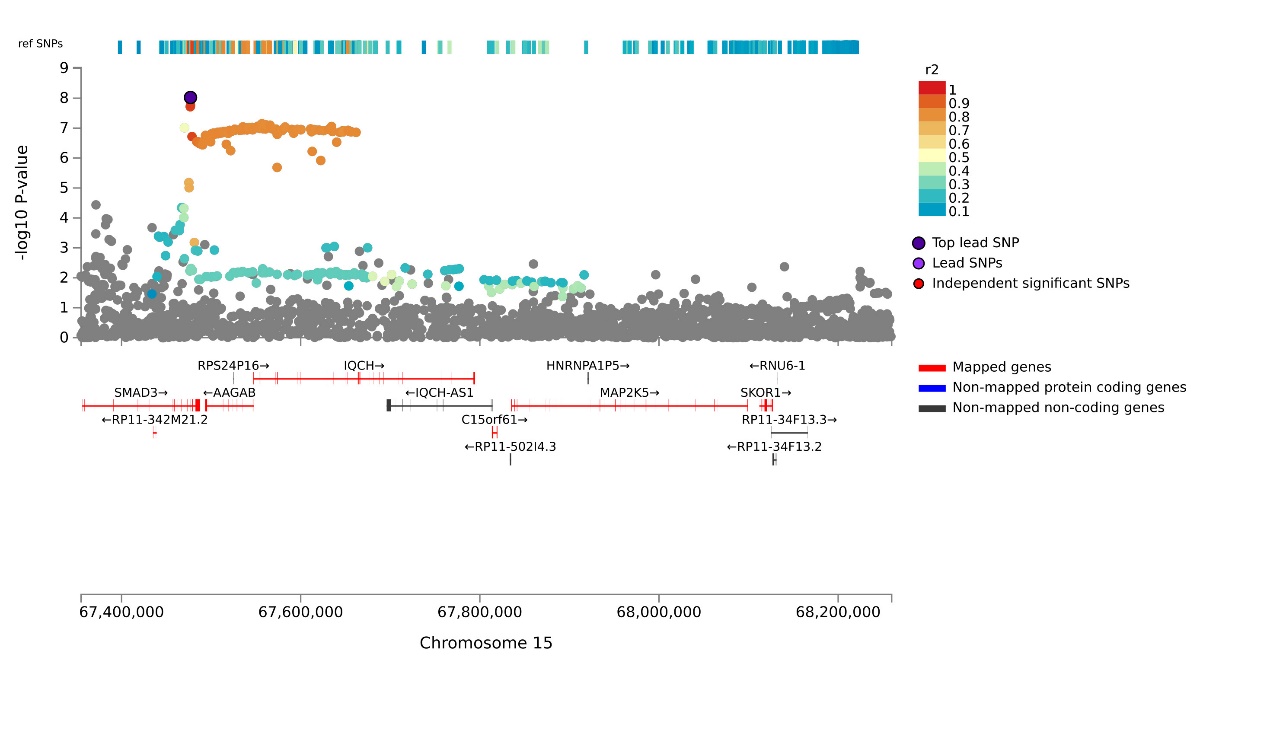


**Figure S10.** The regional plot depicting the shared genetic locus for ASTHMA and EAC, with specific attention to rs12923849.


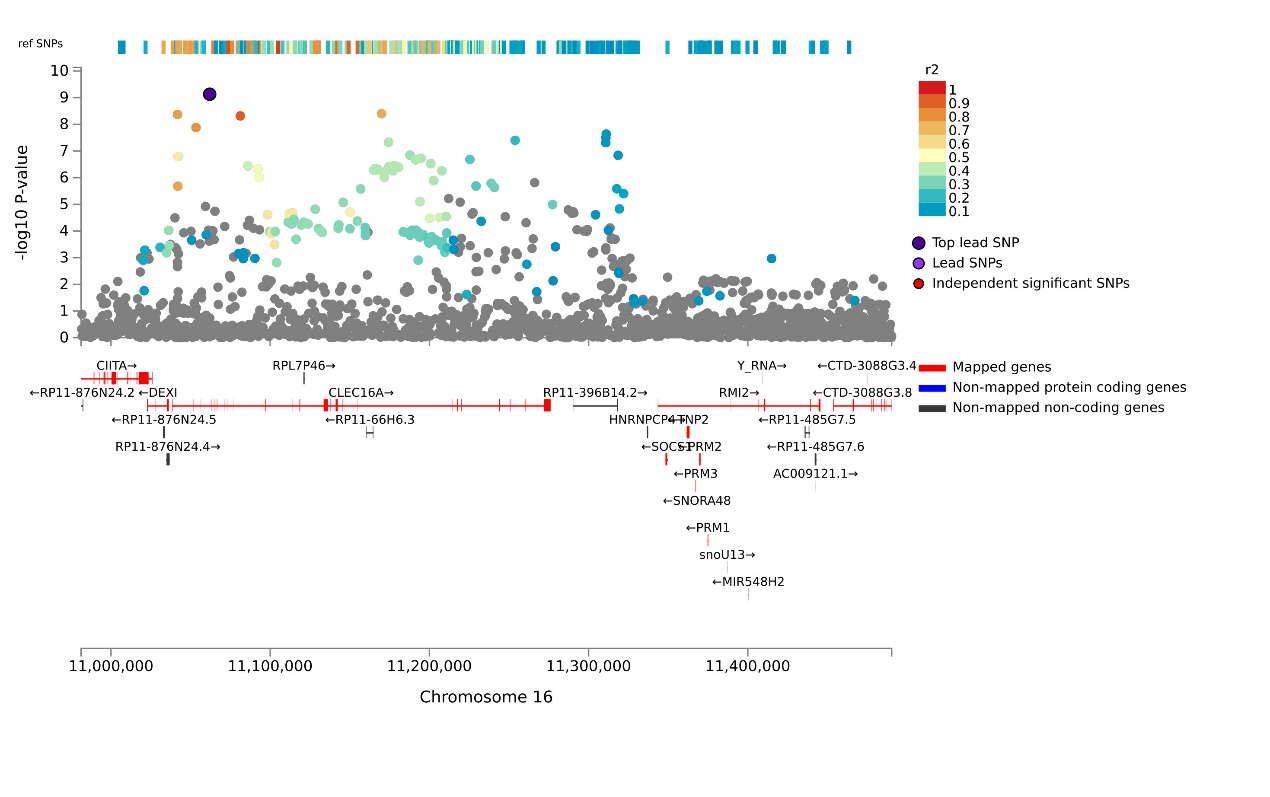


**Figure S11.** The regional plot showcasing the shared genetic locus for ASTHMA and EAC, particularly highlighting rs1558902.


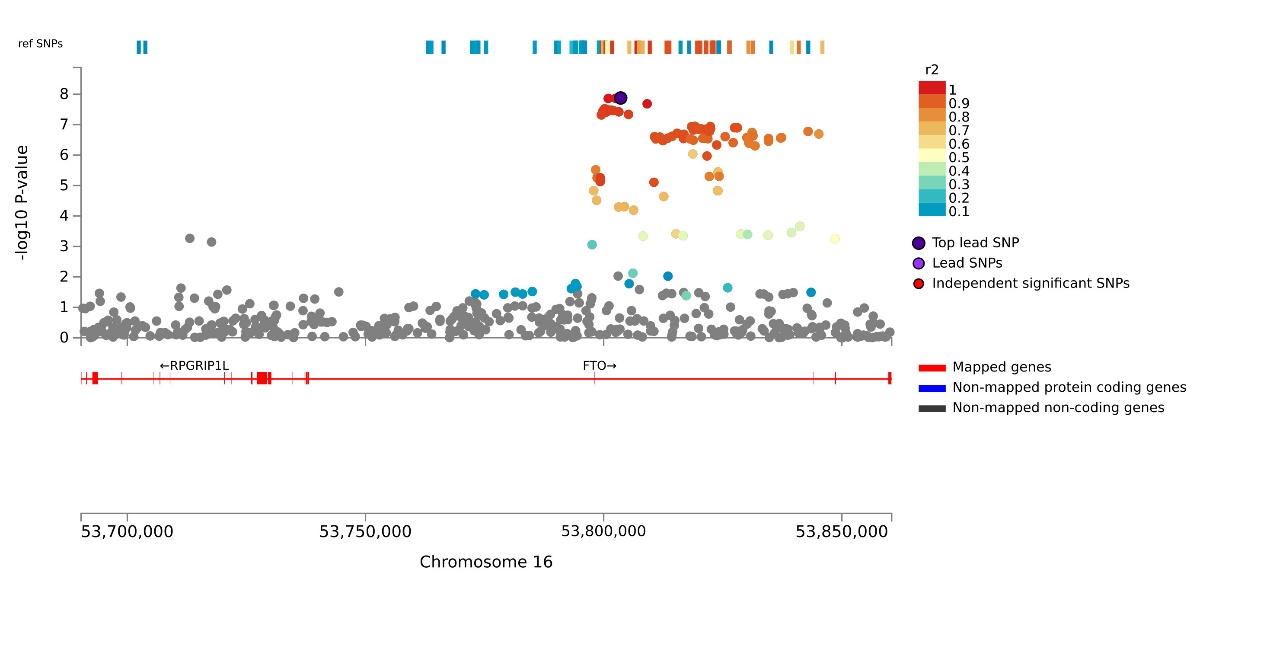


**Figure S12.** The regional plot illustrating the shared genetic locus for ASTHMA and EAC, with a specific focus on rs7256524.


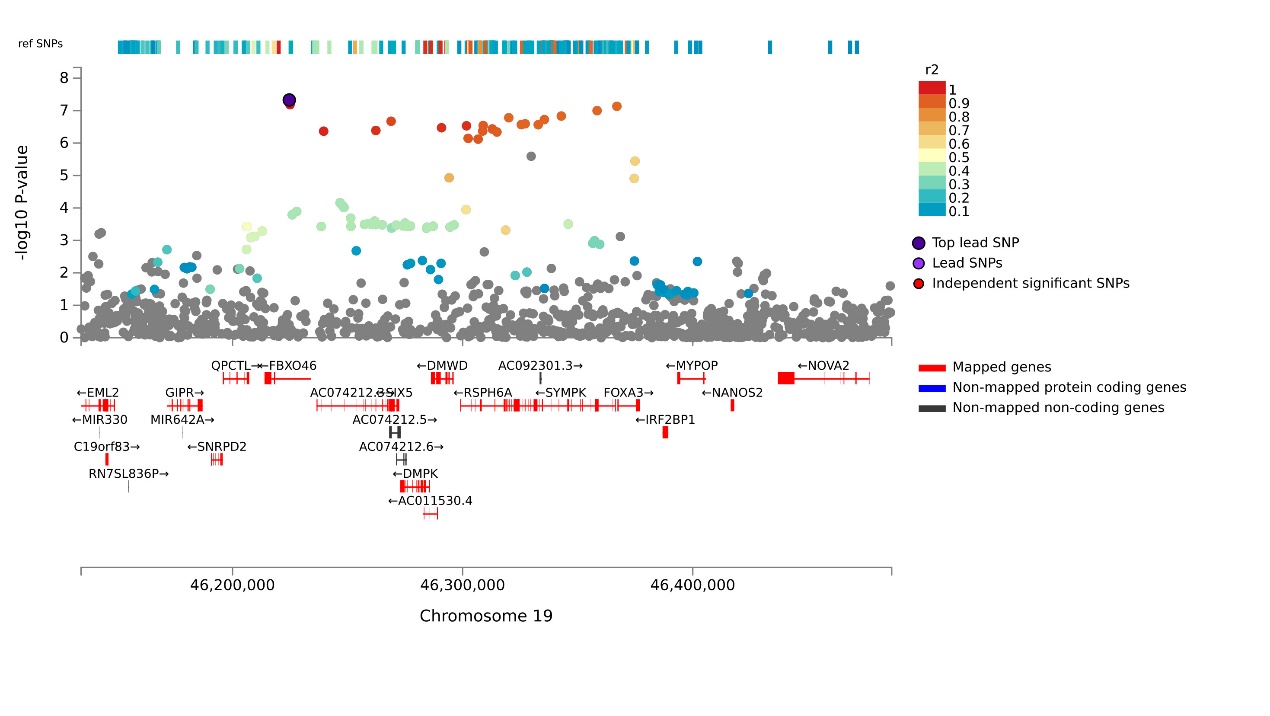


**Figure S13.** The regional plot delineating the shared genetic locus for COPD and EAC, with specific attention to rs13078090.


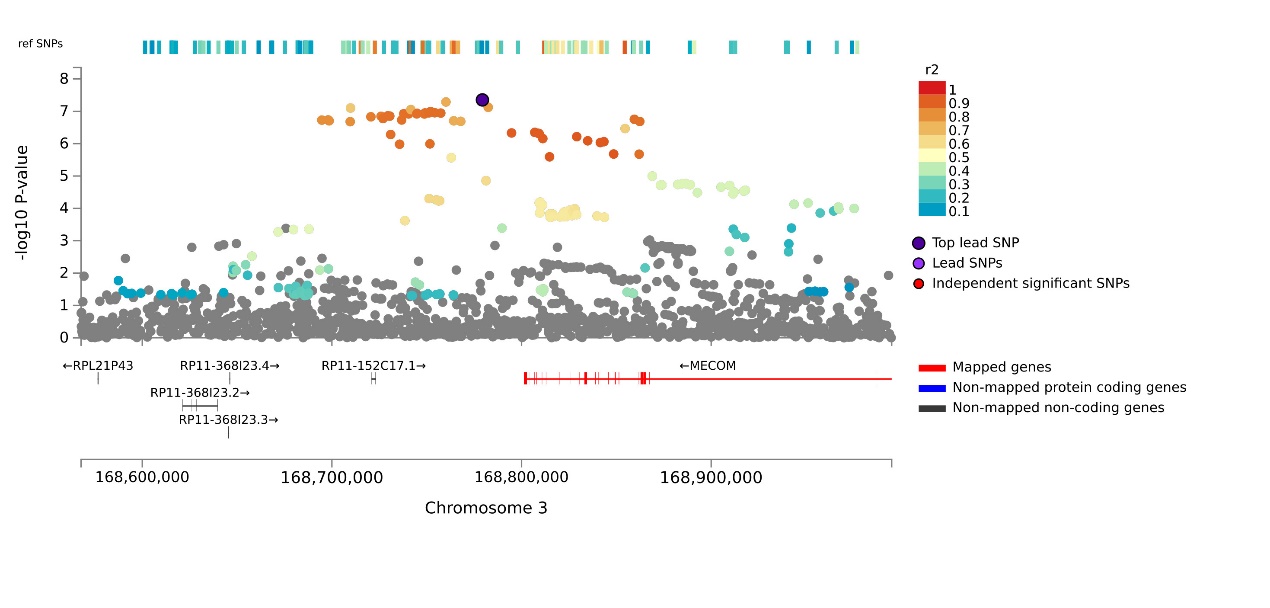


**Figure S14.** The regional plot illustrating the shared genetic locus for COPD and EAC, with specific emphasis on rs1032296.


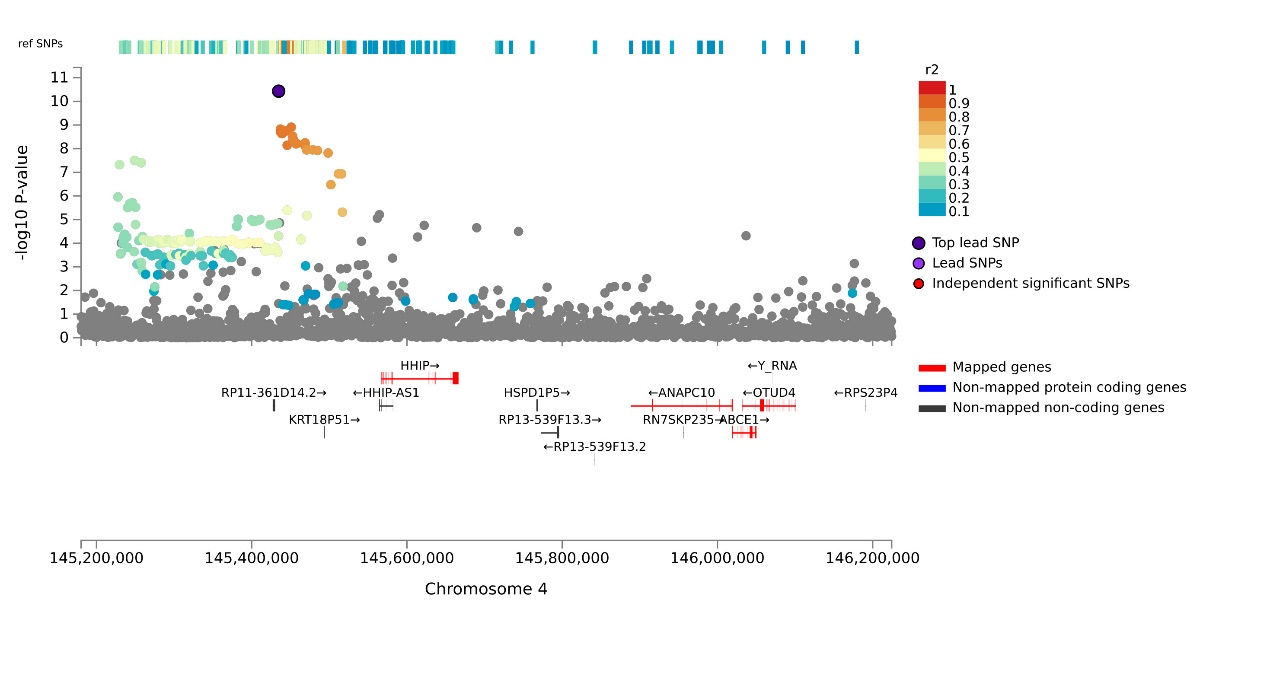


**Figure S15.** The regional plot depicting the shared genetic locus for COPD and EAC, specifically focusing on rs10278953.


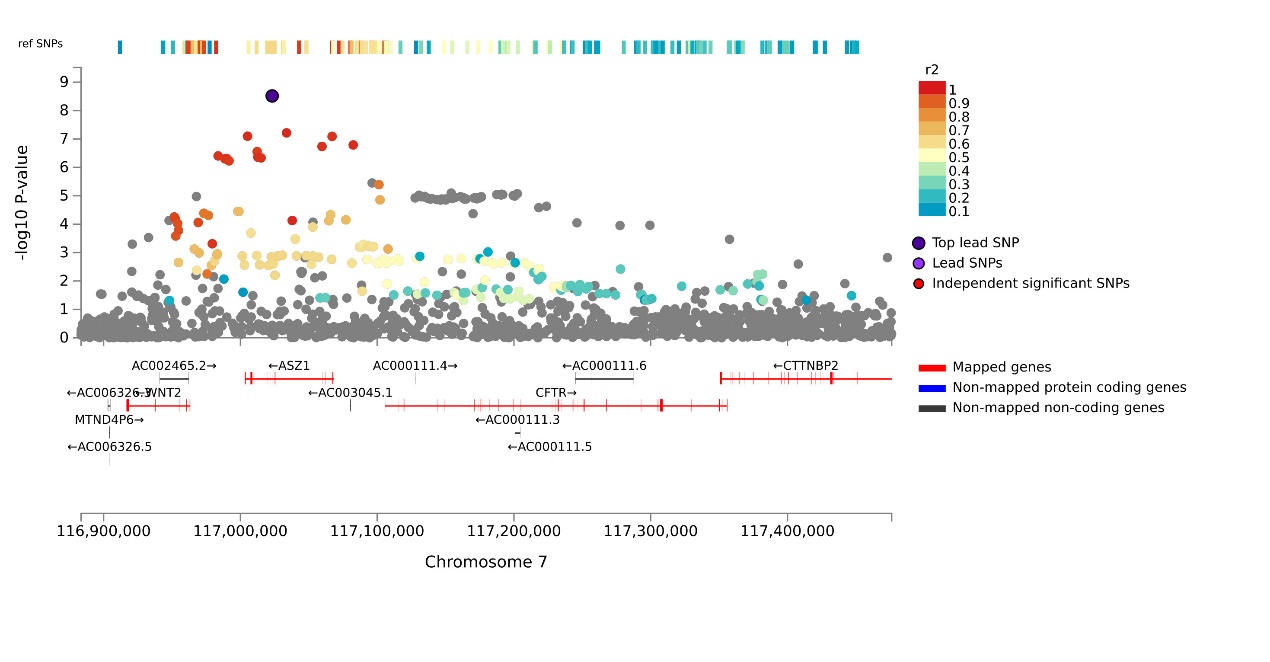


**Figure S16.** The regional plot delineating the shared genetic locus for COPD and EAC, particularly highlighting rs564585.


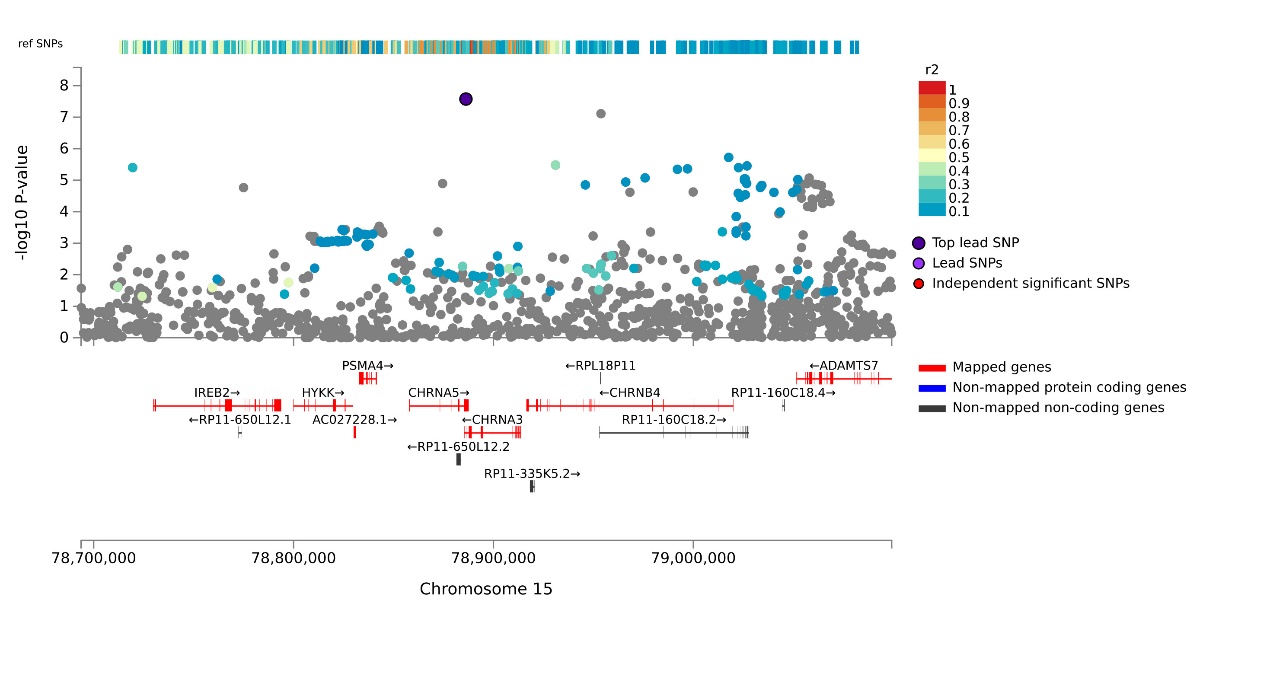


**Figure S17.** The Manhattan plot depicting the results of the MAGMA gene analysis.


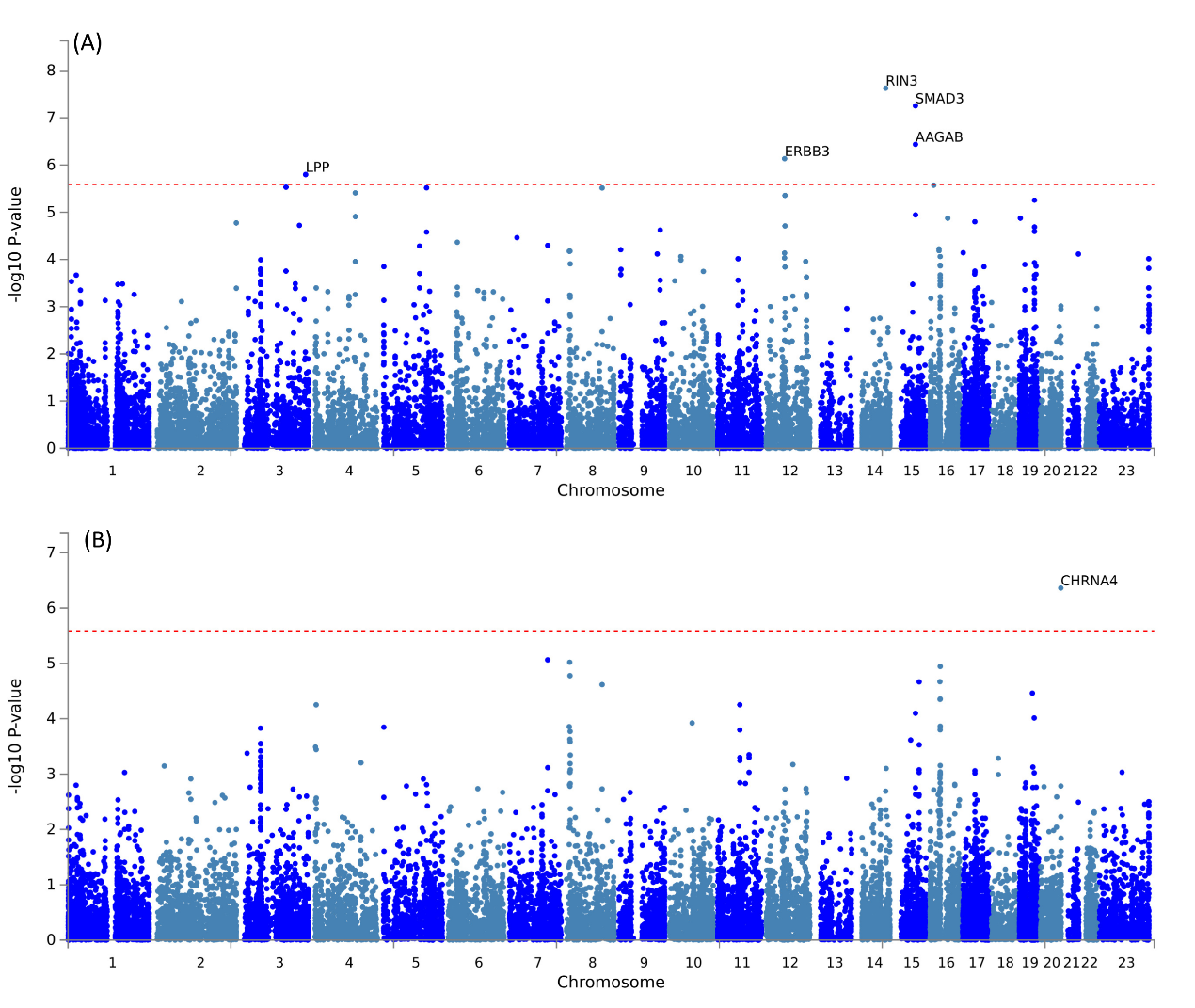


**Figure S18.** The QQ plot illustrating the MAGMA gene analysis results.


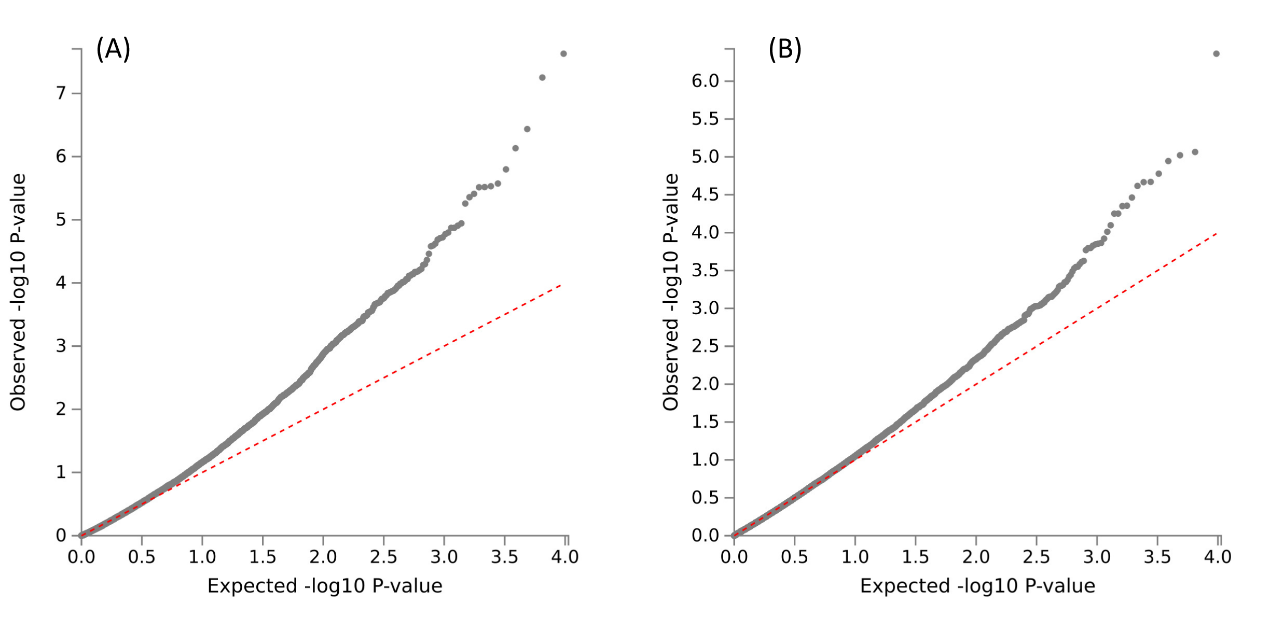


**Figure S19.** The expression profile of pleiotropic MAGMA genes across various tissues.


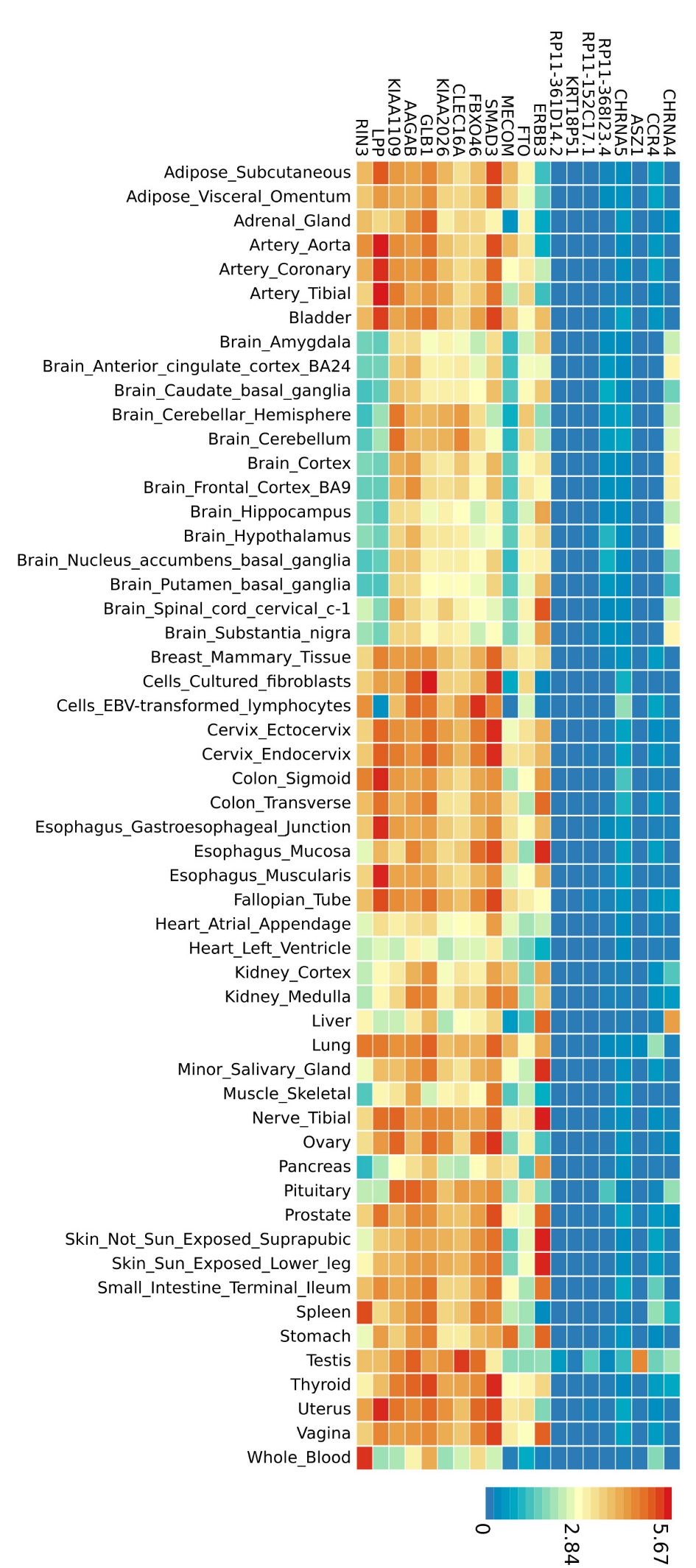


**Figure S20.** The expression patterns of pleiotropic MAGMA genes in different tissues.


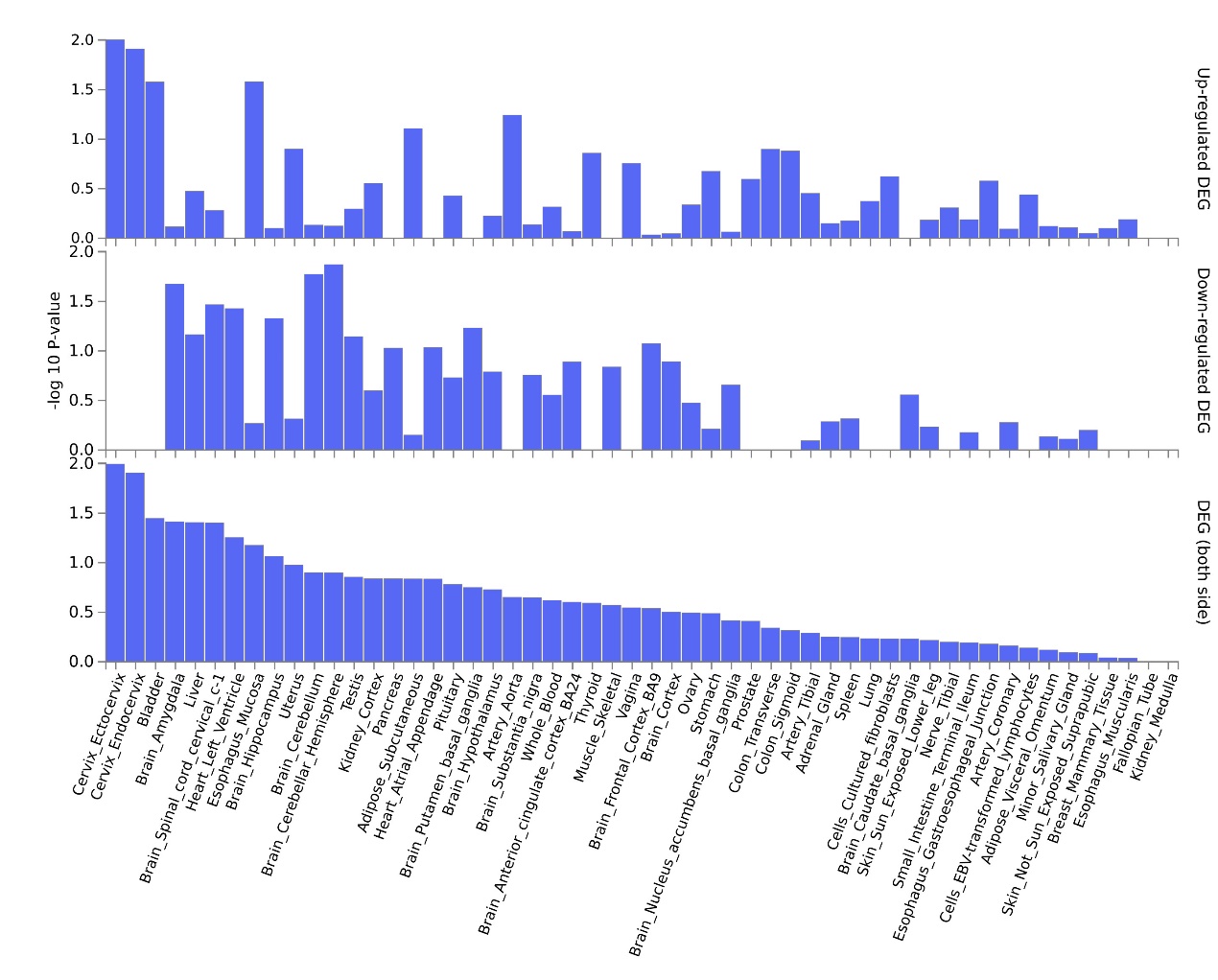


**Figure S21.** The pathway enrichment status of pleiotropic MAGMA genes in different tissues, specifically focusing on Gene Ontology Molecular Function (GO: MF) pathways.


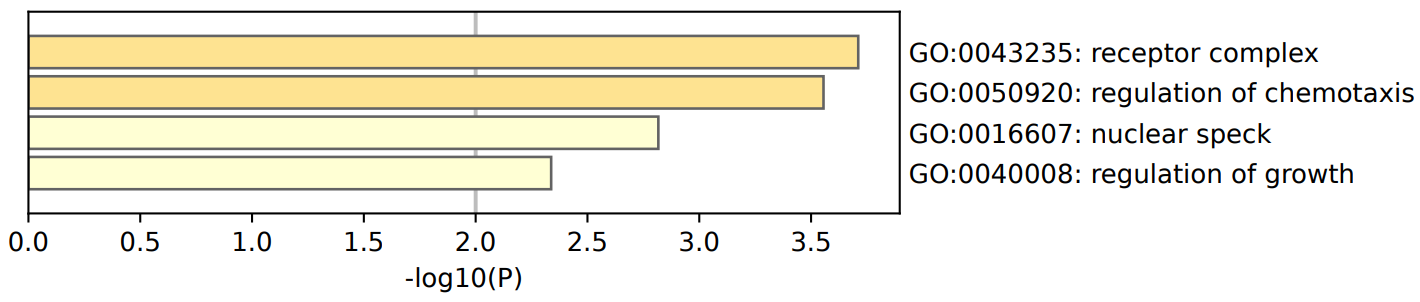


**Figure S22.** The enrichment status of pleiotropic MAGMA genes in immune-related pathways.


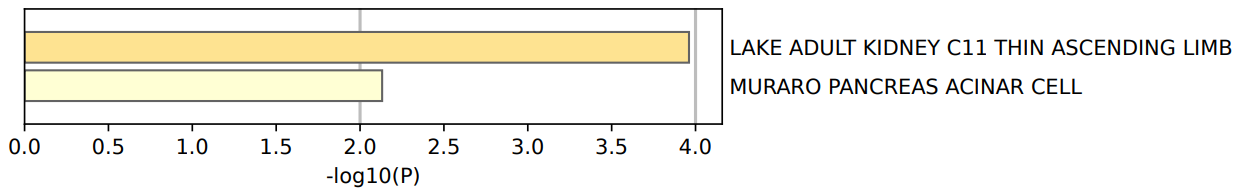


**Figure S23.** The pathway enrichment status of pleiotropic eQTL genes.


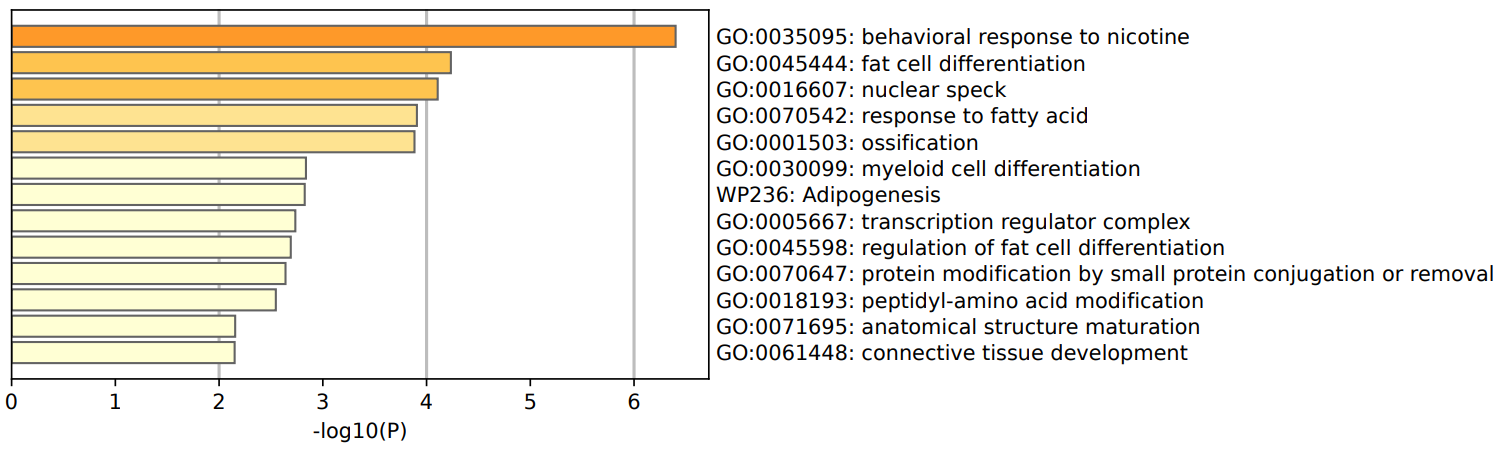


**Figure S24.** The protein-protein interaction (PPI) network analysis of pleiotropic eQTL genes.


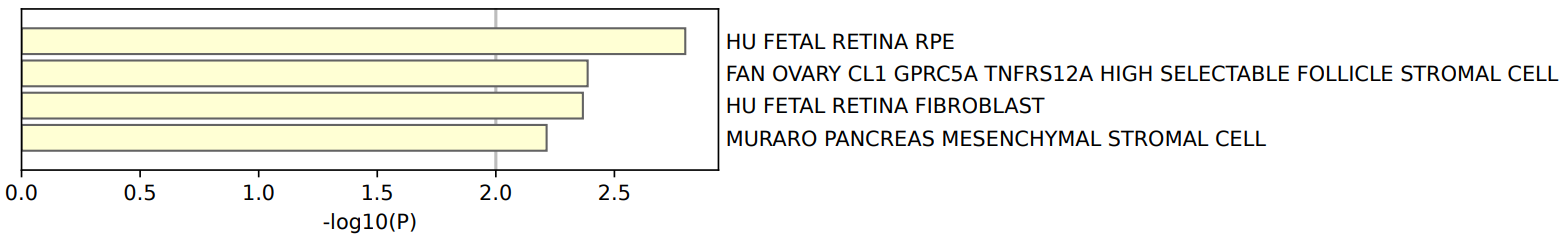

Supplement: Supplementary file 1 — Supplementary figures and tables. [file jcav15p3406s1.zip › Figure S.docx]
